# Presence of Infected Gr-1^int^CD11b^hi^CD11c^int^ Monocytic Myeloid Derived Suppressor Cells Subverts T Cell Response and Is Associated With Impaired Dendritic Cell Function in *Mycobacterium avium*-Infected Mice

**DOI:** 10.3389/fimmu.2018.02317

**Published:** 2018-10-16

**Authors:** Ketema Abdissa, Andreas Nerlich, Andreas Beineke, Nanthapon Ruangkiattikul, Vinay Pawar, Ulrike Heise, Nina Janze, Christine Falk, Dunja Bruder, Ulrike Schleicher, Christian Bogdan, Siegfried Weiss, Ralph Goethe

**Affiliations:** ^1^Institute for Microbiology, University of Veterinary Medicine Hannover, Hannover, Germany; ^2^Department of Molecular Immunology, Helmholtz Centre for Infection Research, Braunschweig, Germany; ^3^Institute for Pathology, University of Veterinary Medicine Hannover, Hannover, Germany; ^4^Mouse Pathology, Helmholtz Centre for Infection Research, Braunschweig, Germany; ^5^Institute of Transplant Immunology, Hannover Medical School, Hannover, Germany; ^6^Immune Regulation Group, Helmholtz Centre for Infection Research, Braunschweig, Germany; ^7^Institute of Medical Microbiology and Hospital Hygiene, Otto-von-Guericke University Magdeburg, Magdeburg, Germany; ^8^Mikrobiologisches Institut - Klinische Mikrobiologie, Immunologie und Hygiene, Friedrich-Alexander-Universität Erlangen-Nürnberg, Universitätsklinikum Erlangen, Erlangen, Germany; ^9^Medical Immunology Campus Erlangen, FAU Erlangen-Nürnberg, Erlangen, Germany; ^10^Institute of Immunology, Hannover Medical School, Hannover, Germany

**Keywords:** MDSC, non-tuberculous mycobacteria, T cells, dendritic cells, iNOS, Nos2, Arg1

## Abstract

Myeloid-derived suppressor cells (MDSC) are immature myeloid cells with immunomodulatory function. To study the mechanism by which MDSC affect antimicrobial immunity, we infected mice with two *M. avium* strains of differential virulence, highly virulent *Mycobacterium avium* subsp. *avium* strain 25291 (MAA) and low virulent *Mycobacterium avium* subsp. *hominissuis* strain 104 (MAH). Intraperitoneal infection with MAA, but not MAH, caused severe disease and massive splenic infiltration of monocytic MDSC (M-MDSC; Gr-1^int^CD11b^hi^CD11c^int^) expressing inducible NO synthase (Nos2) and bearing high numbers of mycobacteria. Depletion experiments demonstrated that M-MDSC were essential for disease progression. NO production by M-MDSC influenced antigen-uptake and processing by dendritic cells and proliferation of CD4^+^ T cells. M-MDSC were also induced in MAA-infected mice lacking Nos2. In these mice CD4^+^ T cell expansion and control of infection were restored. However, T cell inhibition was only partially relieved and arginase (Arg) 1-expressing M-MDSC were accumulated. Likewise, inhibition of Arg1 also partially rescued T cell proliferation. Thus, mycobacterial virulence results in the induction of M-MDSC that block the T cell response in a Nos2- and Arg1-dependent manner.

## Introduction

Non-tuberculous mycobacteria (NTM, also known as ubiquitous or environmental mycobacteria or mycobacteria other than tuberculosis) can lead to serious infections in humans and animals. In recent years, the prevalence and incidence of human NTM infections have increased at an alarming rate, mostly due to the rising number of immunocompromised patients (1–5).

Among the numerous NTM species, *Mycobacterium* (*M*.) *avium* is a paradigm for a pathogenic NTM. It is most frequent cause of infections (1). *M. avium*, which belongs to the *M. avium intracellulare* complex, comprises three major subspecies, *M. avium* subsp. *avium* (MAA), *M. avium* subsp. *hominissuis* (MAH) and *M*. *avium* subsp. *paratuberculosis* (MAP) (6). The *M. avium* subspecies differ strongly in their host range, virulence and tissue tropism (6, 7). MAA causes tuberculosis in birds and is a potential zoonotic and opportunistic pathogen in humans (7). MAP is the well-known causative agent of Johne's disease, a chronic fatal enteritis of ruminants (8). MAH can cause systemic disease in immunocompromised as well as localized disease in immunocompetent humans (9, 10). All *M. avium* subspecies are known to elicit chronic infections and granuloma formation in inbred mouse models (11). However, degree and outcome of such infections vary between subspecies and individual bacterial strains (11–13).

It is now well established that pathogenic mycobacteria not only reside in macrophages, but also in other phagocytes including myeloid derived suppressor cells (MDSC). MDSC represent a heterogeneous population of immature myeloid cells. They are broadly characterized by co-expressing the myeloid lineage differentiation antigen Gr-1 (also known as Ly6C/G) and CD11b (also known as α_M_-integrin). MDSC can be further subdivided into polymorphonuclear MDSC (PMN-MDSC; CD11b^+^Ly6G^+^Ly6C^−^) and monocytic MDSC (M-MDSC; CD11b^+^Ly6G^−^Ly6C^hi^) (14). M-MDSC usually lack surface markers of inflammatory monocytes such as CD11c and MHC class II (15, 16). In mice, normal bone marrow contains 20–30% of cells with MDSC phenotype. In contrast, only a low number is found in naive spleen (2–4%) and they are absent from lymph nodes (17). The number of MDSC can dramatically expand during pathologic conditions, like cancer or infection (17).

MDSC use a plethora of effector molecules to regulate innate and adaptive immune responses. Most commonly employed are inducible nitric oxide (NO) synthase (Nos2) and arginase 1 (Arg1) (14, 17). NO might be the major effector molecule to attenuate immune responses during inflammation and cancer (18, 19). On the other hand, local production of microbicidal NO at the site of infection was also shown to control various viral, bacterial and parasitic pathogens (20–22).

Early studies already reported the generation of T cell-suppressing macrophages during the course of tuberculosis in humans. Similarly, suppressor macrophages were found in mice experimentally infected with M. *tuberculosis* (MTB) or NTM species (23–26). Today, these cells are defined as MDSC and have been described in MTB as well as other bacterial infections (27, 28). Despite several previous reports, the role and phenotype of MDSC in mycobacterial infection is still not completely understood and controversies exist (29).

In the present study we dissected the role of M-MDSC in *M. avium*-infected C57BL/6 mice that are usually able to control mycobacterial infection. Large numbers of NO-producing M-MDSC were induced which were found to be responsible for disease aggravation. These M-MDSC used NO and Arg1 to subvert the immune response in MAA-infected mice by directly inhibiting T cell proliferation and indirectly by a novel mechanism, i.e., by impeding antigen-processing and presentation by conventional dendritic cells. Together, our data for the first time clearly demonstrate that the induction of Nos2- and Arg1-expressing M-MDSC provides an immune escape mechanism that supports infection and pathology by virulent *M. avium* subspecies.

## Materials and methods

### Mice

Female C57BL/6J mice were purchased from Janvier (La Genest-saint-Isle, France) and maintained under specific pathogenic free conditions (SPF) at the animal facility of the Helmholtz Centre for Infection Research (HZI), Braunschweig, Germany. *Thy1.1* OVA albumin transgenic II (OT-II) C57B6L/J mice were bred at HZI. Nos2^−/−^ C57BL6/J mice were obtained from the breeding facility of the Universitätsklinikum Erlangen. Mice were infected at the age of 7–12 weeks. In all experiments mice were placed on *ad-libitum* feeding and unlimited access to water. Mice were maintained for maximum of 5 weeks after infection. All animals experiments were conducted in accordance with the German law for animal protection (Tierschutzgesetz). Approval of study was granted from research ethics committee of the local authority LAVES in Lower Saxony (permission No. 3392 42502-04-13/1192).

### Growth of mycobacterial strains and infection

Bacterial strains for infection were grown in Middlebrook 7H9 broth. Mice were infected intraperitoneally (i.p.) by injection of 200 μl (~10^8^ CFU) of the bacterial suspension. Bacterial strains (*M. avium* ATCC 25291 and *M. avium* 104) were grown in Middlebrook 7H9 broth (BD) supplemented with 0.5% glycerol and 10% OADC. To attain early logarithmic phase of bacterial growth, broth was inoculated with mycobacteria to an optical density 600 (OD_600nm_) of 0.2 and cultures were grown at 37°C under stirring (130 rpm) until a final OD of ~1. The bacterial culture was washed 3 times with Dulbecco's phosphate buffered saline (DPBS). To avoid bacterial clumping, the suspension was intensively vortexed including 3 mm glass beads. Bacterial suspension was adjusted to OD_600_ of 5 in DPBS for infection. In addition, the same OD of heat inactivated bacteria (85°C for 15 min) was applied in some experiments.

### Flow cytometry and cells sorting

Spleen cell suspensions were prepared by gently flushing the organs with Iscove's complete medium (IMDM, 10% heat inactivated fetal calf serum, penicillin 100 unit/ml, streptomycin 100 μg/ml, 2 mM L-glutamine, 50 μM 2-mercaptoethanol). Then cells were filtered through 70 μm and finally through 50 μm diameter cell strainers. Red blood cells were removed by erythrocyte lysis buffer (14.2 mM sodium hydrogen carbonate (NaHCO_3_), 155 mM ammonium chloride (NH_4_Cl), 0.1 mM EDTA, at final pH of 7.3). Cells were stained in FACS buffer (PBS containing 2 mM EDTA, 2% FBS). Anti-CD16/32 (clone 2.4G2, FCR block) was applied before staining with specific antibodies. Dead cells were excluded with DAPI staining. Separate staining was done for each fluorochrome conjugated antibody to determine positive and negative cell populations. All antibodies used, with their respective clones are indicated in Table S1. Data were acquired on LSR II analyzer (BD, NJ, USA). Data analysis was done using FACSDiva software (BD) or FlowJo (TreeStar). Cell sorting was done on BD FACSAria-II. Re-analysis of sorted cells was done for purity check. The gating strategy is provided in Figure S5.

### Intracellular staining

For intracellular staining, 10^7^ spleen cells were incubated in 200 μl IMDM containing 5 μg brefeldin A (BioLegend) for 2 h at 37°C. Cells were stained for surface markers using standard staining protocol. Then cells were fixed in fix/perm buffer (BD), stained for intracellular markers in perm/wash buffer (BD) and analyzed using flow cytometry.

### *Ex vivo* antigen dependent T cell proliferation

Spleen conventional dendritic cells, CD11c^hi^CD11b^+/−^ (cDC) from spleen were sorted and pulsed with Endograde ovalbumin protein (Hyglos, 100 μg/ml) and ovalbumin peptide (Aa _323−339_, 1 μg/ml) for 1 h at 37°C in IMDM. CD4^+^ T cells were isolated (>90% purity) from OT-II spleen using Dynabeads® Untouched™ Mouse CD4^+^T Cells Kit (Invitrogen). Cells were stained with 5 μM carboxyfluorescein diacetate succinimidyl ester (CFSE) (Invitrogen) and incubated for 10 min at 37°C and washed 3 times in complete medium. Finally viable 3 × 10^4^ DC were co-cultured with viable 3 × 10^5^ CD4^+^ T cells in complete medium. Percentage of cells that divided at least once was determined after 3 days for peptide or 4 days for protein.

### *Ex vivo* T cell proliferation assay

Untouched CD4^+^ T cells were purified from infected and PBS control mice spleen. Isolated cells were stimulated (2 × 10^5^ cells/well) with either plate bound anti-CD3 (5 μg/ml, clone 45-2c11) or in combination with soluble anti-CD28 (500 ng/ml, clone 37.51). Four days after stimulation, percentage proliferation of cells was analyzed by CFSE dilution.

### Nos2 dependent *ex vivo* T cell inhibition assay

Infection induced spleen M-MDSC were sorted and co-cultured with naïve, CFSE labeled CD4^+^ T cells in the presence of immobilized anti-CD3 antibody (5 μg/ml). Different ratios of T cells and M-MDSC were tested in the presence or absence of Nos2 inhibitor, L-NIL (Cayman chemicals) at a final concentration of 40 μM. T cells proliferation was measured after 4 days in culture.

### *In vivo* T cell proliferation

Naïve CFSE labeled *Thy1.1* OT-II CD4^+^ T cells (2 × 10^6^) were injected intravenously via lateral tail vein. After 24 h, 200 μg ovalbumin protein was injected i.p. Three days after immunization, mice were sacrificed and spleen was collected. *In vivo* proliferation of *Th1.1* expressing CD4^+^ T cells was monitored by CFSE dilution using flow cytometry. The gating strategy is provided in Figure S5B.

### Antigen uptake and processing assay

A standard antigen uptake (OVA-cy5 endocytosis) and processing (DQ-OVA degradation) protocol was followed (30). Briefly, cDC were incubated with 100 μg/ml OVA-cy5 or 62.5 μg/ml DQ-OVA for 90 min and analyzed using flow cytometry. To determine the rate and compartment in which degradation of DQ-OVA takes place in cDC, spleen cDC were sorted and seeded on poly-L-lysine (Life science, Sigma Aldrich) coated coverslip at a density of 2 × 10^5^ cells per well overnight. After changing to new complete medium, cells were pulsed with 62.5 μg/ml DQ-OVA and incubated for 45 min at 37°C. After 3x washings, cells were further incubated for 2 h at 37°C. Then cells were washed and fixed in 20% eBioscience™ fixation buffer. Fixed cells were stained with lysosome associated membrane protein 1 (LAMP-1) antibodies (BioLegend). Finally, images were taken by confocal fluorescent microscopy.

### Depletion of Gr-1 expressing cells

Eleven days after infection, 250 μg of anti-Gr-1 antibody (clone RB6-8C5) was given intraperitoneally at day 11, 14 and 17 post infection (p.i.) and mice were sacrificed on day 20. Control mice were injected with similar concentration of rat IgG (Dianova). Spleen cells were prepared for flow cytometry and livers were collected for histology and plating.

### Splenocytes nitrite assay

Spleen single cell suspensions were prepared following the above standard protocol. A total of 10^7^ cells were seeded in 2 ml complete IMDM in 6-well cell culture plates and incubated for 48 h. Cell culture supernatants were collected and nitrite content analyzed using the Griess reagent system (Promega).

### *In vitro* NO susceptibility testing

For testing nitric oxide susceptibility bacteria were harvested at early logarithmic growth and diluted in PBS^−^ to an OD_600nm_ of 0.01. Following 10 μl (~10^4^ CFU) were incubated in PBS^−^ containing two different concentrations of nitric oxide donor S-nitrosoglutathione (GSNO) (Sigma) for 4 h at 37°C. Plating was done on Middlebrook 7H10 agar to compare the CFU with and without treatment. The relative survival was calculated referring the number of CFU from bacteria incubated in the presence of the NO donor to that of the input CFU.

### Quantification of intracellular bacteria by PCR

Whole cell (eukaryotic and bacterial) DNA was extracted from sorted and 3% paraformaldehyde fixed cells. Briefly, zirconium beads were added and cells were disrupted using a tissue homogenizer. The homogenate was sonicated using Branson sonifier 450. Supernatant was collected after centrifugation. After adding an equal volume of TE buffer, RNA was removed by adding 10 μl RNase A (Roche) followed by a 1 h incubation at 37°C. To reverse the cross link, 15 μl of 4M NaCl was added and incubated for 5 h at 65°C. Finally, DNA was extracted using standard phenol chloroform extraction method. Bacterial DNA PCR was performed using the IS901 specific primers and normalized against eukaryotic *Cxcl2* (*Mip2a*) promoter. IS901: for_GTGATCAAGCACCTTCGGAA, rev_GCTGCGAGTTGCTTGATGAG; *Mip2a*: for_GAAGGGCAGGGCAGTAGAAT, rev_ ATGGCGCTAGGCTGAAGTG.

### Quantitative real time PCR

After spleen cells sorting, cells were kept in 500 μl (1X concentrate) DNA/RNA Shield™ (Zymo Research). Following RNA extraction was processed using Direct-zol™ RNA Miniprep kit (Zymo Research). RNA was reverse transcribed using M-MLV transcriptase (Promega) and oligo-(dT) _12−18_ primers (Roth). Quantitation of expression of selected genes was done using TaqMan® Gene Expression Assays (Applied Biosystems) (assay ID; IFNγ: Mm01168134_m1, IL-10: Mm00439616_m1, IL-6: Mm00446190_m1). Primers used for SYBR based expression assays were *Tnf* : for_ ATGAGCACAGAAAGCATGATC rev_TACAGGCTTGTCACTCGAATT; *Il1b*: for_ TTGACGGACCCCAAAAGATG, rev_AGAAGGTGCTCATGTCCTCA; *Nos2*: for_ CCCAGCACAAAGGGCTCAAA, rev_GCACCTGGAACAGCACTCTC; *Arg1:* for_ GATGTCCCTAATGACAGCTCC, rev_AGCACCACACTGACTCTTCC; *Rps*9: for_CTGGACGAGGGCAAGATGAAGC, rev_TGACGTTGGCGGATGAGCACA. Relative gene expression was calculated using housekeeping gene *Rps9* as standard as described before (31).

### ELISA

Blood was collected via cardiac puncture in 500 Serum-Gel tubes (Sarstedt). Serum was separated by centrifugation at 10,000 rpm for 4 min at room temperature and kept at −80°C until analysis. Serum concentration of IL-6 and CCL5 were determined by using PEPROTECH kit following the manufacturer's kit protocol. In addition serum concentration of cytokines and chemokines were quantified by LUMINEX based mouse cytokine 23-plex assay following manufacturer's instruction (Bio-Rad, USA).

### Histopathology

Upon isolation, specimens were fixed with 4% (v/v) formalin and embedded in paraffin. Approximately 3 μm thick sections were cut and stained with hematoxylin/eosin according to standard laboratory procedures. Immuno-histo-chemical staining was performed to detect cleaved caspase-3 (Asp175), using the 3,3′-diaminobenzidine, Zytomed Systems DAB530 as chromogen. Hematoxylin was used for counterstaining. Immunofluorescence staining was performed using following primary antibodies: polyclonal rabbit anti-mouse Nos2 (eBioscience), polyclonal goat anti-human Arg1 (Santa Cruz Biotechnology), anti-mouse MAC-2 (Biozol diagnostics) and self-produced anti-heparin binding hemagglutinin (HBHA). Alexa Fluor anti-rabbit/goat 488 and Alexa Fluor anti-rabbit 594 were used as secondary antibodies. Sections were analyzed by light blinded to the experimental groups.

### Ziehl-Neelsen (ZN) staining

Sorted spleen cells were fixed in 3% paraformaldehyde on ice for 10 min. Fixed cells were suspended in FACS buffer. Fifty micro liter of this cell suspension was spun on the glass slide using a cytospin centrifuge. ZN staining was done following a standard protocol.

### Immunoblot analysis

Splenic myeloid cells were sorted from naïve and infected mice into CD11b^+^CD11c^int^, CD11b^+^CD11c^−^ cell populations according to CD11b and CD11c expression. Cell lysates were prepared in 40 mM Tris buffer (pH 8.0) including 1mM PMSF and a protease inhibitor mix (cOmplete mini EDTA-free, Roche). After sonication, cell lysates were separated on 10% SDS-PAGE and transferred to PVDF membrane (0.45 μm, Millipore Immobilon-P, 1 h, constant current of 1 A) using tank block technique. Nonspecific binding sites were blocked by blocking buffer (5% dry milk, 0.1% Tween 20 in PBS) for 1 h. Target proteins were stained by primary antibodies (goat anti-Arg1, clone V-20, Santa Cruz Biotechnology; mouse anti-GRB2, clone 81/GRB2, BD Biosciences) and HRP conjugated secondary antibodies (donkey anti-goat, goat anti-mouse, both Dianova). Reaction signals were detected by ECL based chemiluminescence. Images were processed with help of the Adobe Photoshop CS5-software (Adobe Systems, San José, CA, USA).

### Statistical analysis

Data were entered into graph pad prism version 5 software. Mean ± standard error of the mean (Mean ± SEM) was used for data description. Statistical test between two groups was determined using Student's *t*-test. Difference between more than two groups was determined either with one way or two way analysis of variance (ANOVA) using Dunnett's multiple comparisons against the PBS control group. One way ANOVA Bonferroni's multiple comparison tests has been used in some figures. CFU counts were compared by Mann Whitney U test. Cut off *p* < 0.05 was considered as statistically significant difference (^*^p < 0.05, ^**^p ≤ 0.01, ^***^p < 0.001).

## Results

### Chronic MAA infection induces accumulation of mycobacteria harboring histiocytic cells in murine spleen

**Figure 1:**
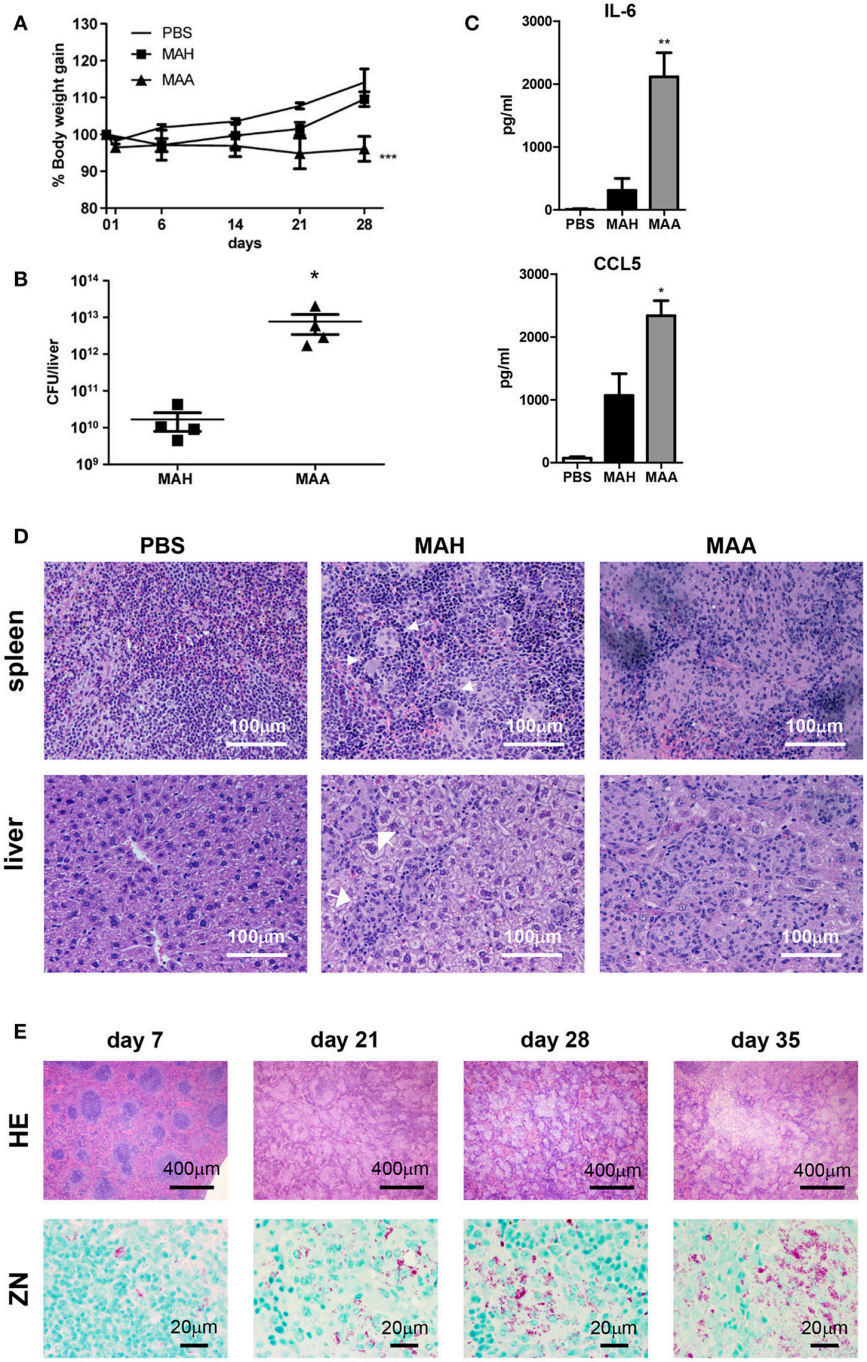
Chronic MAA infection induces accumulation of mycobacteria harboring histiocytic cells in murine spleen. **(A)** Body weight of female C57BL/6J mice infected with MAA or MAH (Mean+SEM, ^***^p < 0.001: two way ANOVA). **(B)** Bacterial load in livers of mice infected with MAA or MAH 5 weeks post infection (p.i.). Colony forming units (CFU) were determined by plating of liver lysates. **(C)** Serum cytokine levels 5 weeks p.i. as determined by ELISA. Statistics shown is difference between two strains. **(D)** Hematoxylin/Eosin (HE) staining of spleen and liver of mice infected with MAA or MAH for five weeks. Arrows indicate well defined granuloma surrounded by rim of lymphocytes. **(E)** HE and Ziehl Neelsen (ZN) staining of MAA infected mice spleen at day 7 until day 35. (**A,B,D,E**: 3-5 mice per group. **A,D**: representative of least two independent experiments. **C**: 6 mice for MAA and MAH groups and 3 mice for PBS control) (Mean+SEM, ^*^p < 0.05, ^**^p < 0.005: One way ANOVA).

It is now clear that pathogenic mycobacteria are capable to infect diverse subsets of myeloid cells. To gain more insight into the role of such myeloid cells during progressive *M. avium* infection, we infected mice intraperitoneally with highly virulent *M. avium* subsp. *avium* (strain ATCC 25291), in the following abbreviated as MAA, or with the genetically closely related, but less virulent *M. avium* subspecies *hominissuis* strain 104 (MAH). MAA-infected mice lost body weight upon infection and were not able to regain it during the course of observation. In contrast, MAH-infected mice quickly recovered after an initial weight loss (Figure 1A). We also found high numbers of bacteria (determined as colony-forming units [CFU]) in the liver of MAA-infected mice at 5 weeks p.i., whereas the bacterial burden was roughly 1,000-fold lower in MAH-infected mice (Figure 1B).

In accordance with the differential weight loss induced by the two bacterial strains, significant differences were seen in the blood levels of proinflammatory cytokines. High concentrations of IL-6 and CCL5, both of which are involved in regulating immune cell migration, were detected by ELISA in MAA-infected mice, but not in mice infected with MAH (Figure 1C). Similarly, multiplex analysis revealed differential expression of IL-1β, IL-1α, IL-5, TNF, and CCL3, whereas most other cytokines and chemokines including IFN-γ were comparably upregulated by the two mycobacterial strains (Figure S1A).

Defined granulomas were formed in the spleens of MAH-infected mice. In contrast, diffuse inflammation dominated by histiocytes and loss of defined lymphoid follicles was seen in the spleens of MAA-infected mice (Figure 1D). In addition, classical granuloma harboring mononuclear cells and peripheral lymphocytes were observed in the liver of MAH-infected mice, whereas granuloma with higher numbers of mononuclear cells and low numbers of lymphocytes were found in the livers of MAA-infected mice (Figure 1D).

To study the diffuse histiocytic granulomatous inflammation in more detail, hematoxylin/eosin (HE) and Ziehl-Neelsen (ZN) stainings of the spleens of MAA-infected mice were analyzed at different time points (Figure 1E). At 1 week of infection, there were little changes of splenic structures; lymphoid follicles were clearly visible. However, after 3 weeks the general architecture of the spleen changed completely. Lymphoid follicles disappeared and the progressive granulomatous inflammation with increasing numbers of myeloid cells became apparent. Furthermore, numerous acid-fast bacteria became detectable by ZN staining (Figure 1E). In fact, a 10^5^-fold increase in bacterial CFU numbers between week 1 and week 3 p.i. was observed (Figure S1B).

### Chronic MAA infection induces accumulation of disease-aggravating Gr-1^int^CD11b^hi^CD11c^int^ cells in the spleen

**Figure 2:**
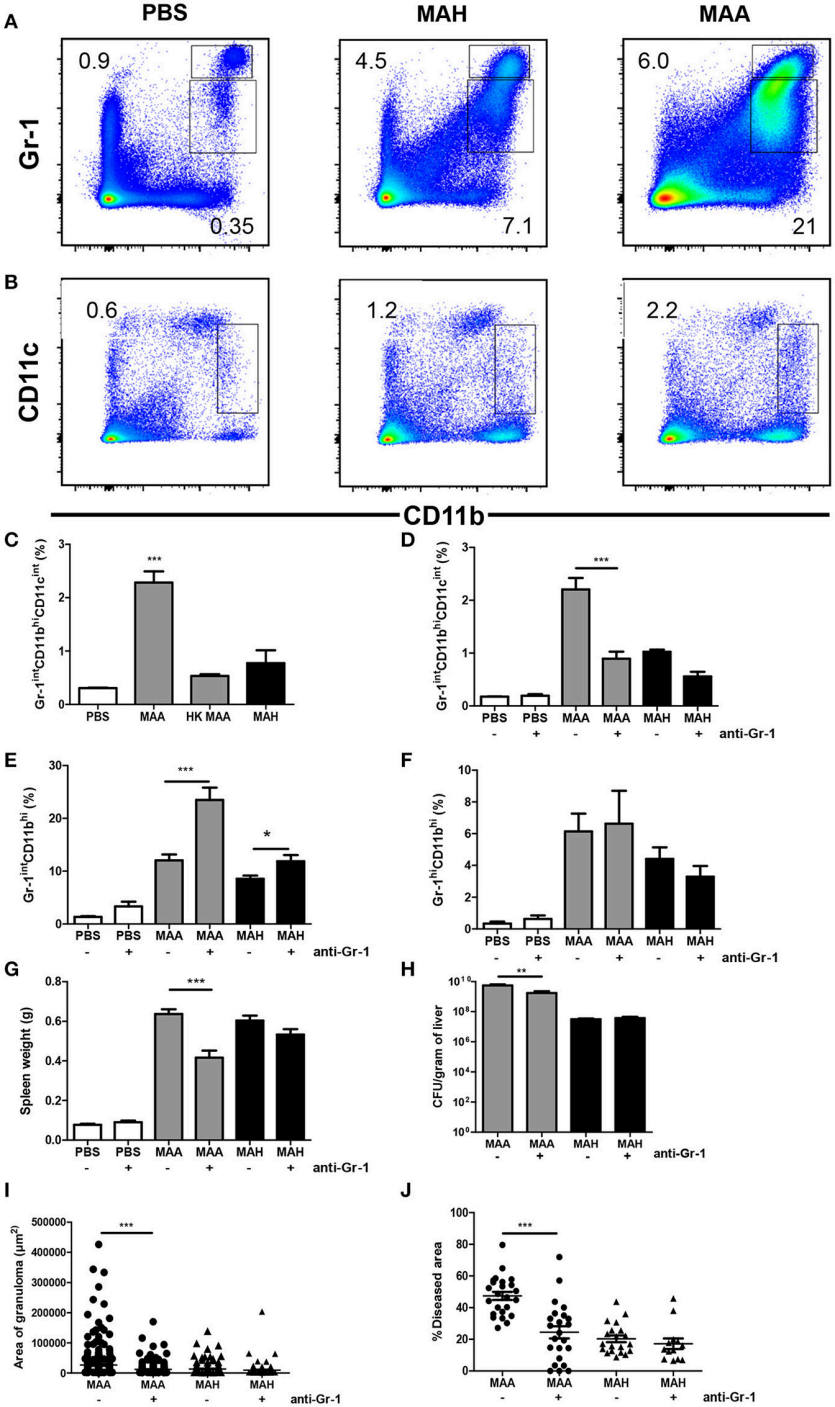
Chronic MAA infection induces accumulation of disease aggravating Gr-1^int^CD11b^hi^CD11c^int^ cells in murine spleen. **(A,B)** Flow cytometry of spleen cell suspensions from MAA or MAH-infected mice showing the percentage of Gr-1^hi/int^ and CD11b^hi^ splenic myeloid cells. **(C)** Percentage of spleen CD11b^hi^CD11c^int^ cells of MAA, HK (heat killed) MAA or MAH-infected mice as determined by flow cytometry (^***^p < 0.001: one way ANOVA). **(D–J)** Mice infected with MAA or MAH were treated with PBS or an anti-Gr-1 antibody according to the schedule described in Material and Methods. Percentage of splenic Gr-1^int^CD11b^hi^CD11c^int^ cells **(D)**, Gr-1^int^CD11b^hi^ **(E)**, and Gr-1^hi^ CD11b^hi^ **(F)**. **(G)** Spleen weights. **(H)** Bacterial load from liver homogenate. **(I,J)** Morphometry of liver pathology shown by the area of granuloma and the percentage of diseased area per 100x magnification of liver section (at least five fields were analyzed per section). Analysis 3.1 software package (Soft Imaging System) was used for quantification. Figure **(A–C)** shows representative of at least two experiments (3-5 mice per group). **(D–J)** represents two independent experiments of 3-5 mice per group. (^*^p < 0.05, ^**^p < 0.01, ^***^p < 0.001: *t*-test).

**Figure 3:**
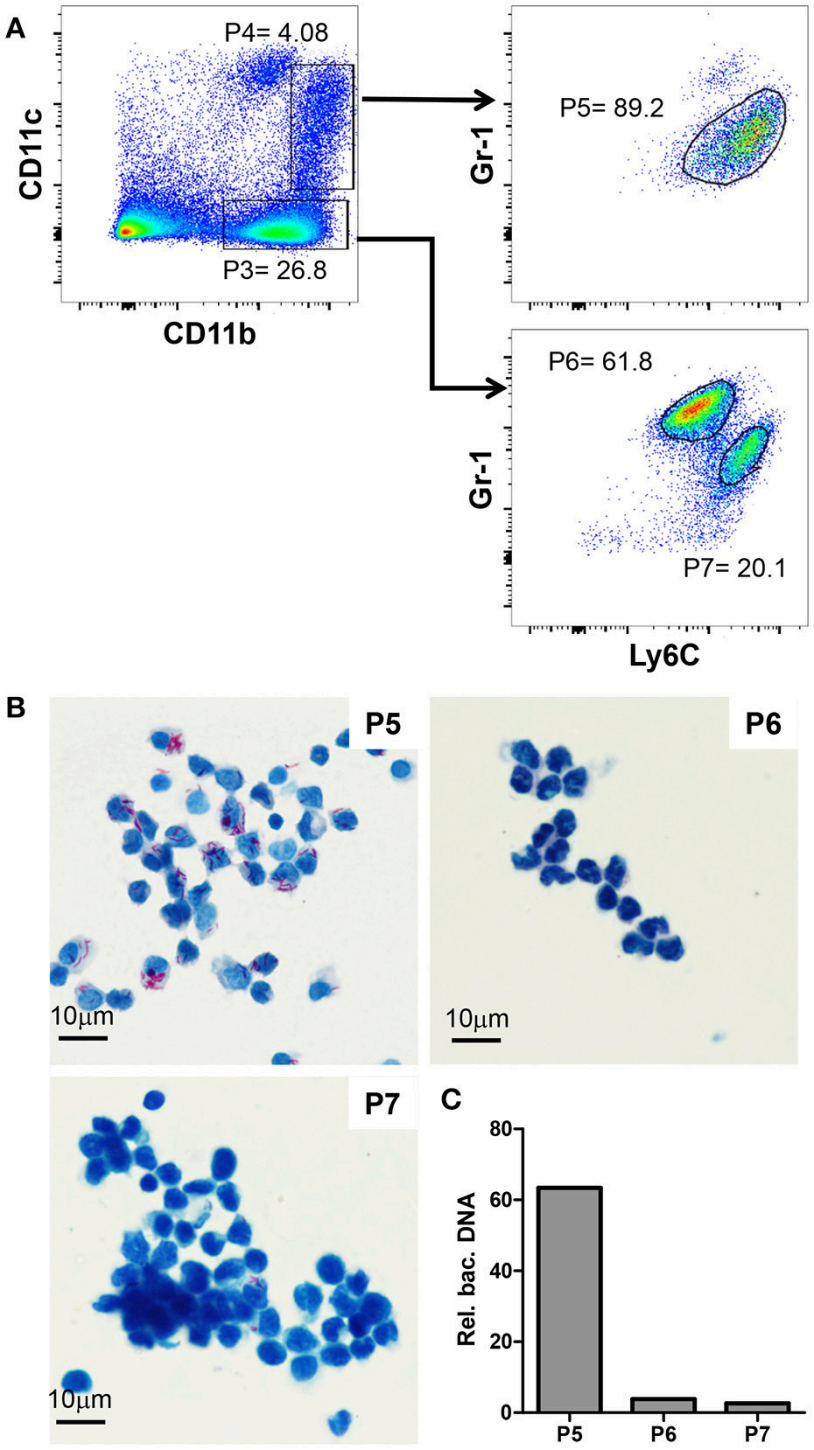
Gr-1^int^CD11b^hi^CD11c^int^Ly6C^hi^ cells are monocytic myeloid derived suppressor cells and heavily infected with MAA. **(A)** Flow cytometry of spleen suspensions from MAA-infected mice showing the percentage of Ly6C^+^ cell among CD11b^hi^CD11c^−^ (P3) and CD11b^hi^CD11c^int^ (P4) cells; gated populations: Ly6G^−^Ly6C^+^Gr-1^int^ (P5) cells, P6 = Ly6C^lo^Ly6G^+^Gr-1^hi^ cells, Ly6^+^Ly6G^−^Gr-1^int^ (P7) cells. **(B)** ZN staining of cytospins of the populations gated in **(A)**. **(C)** Relative abundance of bacterial DNA in the populations gated in **(A)**. Graph shows qPCR analysis of DNA extracted from the cell populations using primers for mycobacterial *IS901* and the mouse *Mip2a* promoter for normalization. Bars show the amount of bacterial DNA relative to mouse DNA. Data from 3-5 animals per group is shown.

To investigate the mechanism underlying the different host reactions in MAA- versus MAH-infected mice, we analyzed the monocytic cell populations in the spleens of infected mice in more detail. MAA and MAH infection induced accumulation of Gr-1^hi^ as well as Gr-1^int^ cells. Especially in MAA-infected mice, the numbers of Gr-1^int^CD11b^hi^ cells were strongly increased compared to Gr-1^hi^CD11b^hi^ cells (Figure 2A, Figures S2A,B). Furthermore an accumulation of CD11b^hi^CD11c^int^ cells was detected in spleens of mice infected by MAA but not in MAH-infected mice or mice treated with heat inactivated MAA (Figures 2B,C, Figure S2C). These cells were Gr-1^int^ (Figure 3A).

The massive influx of myeloid cells was associated with changes in the lymphoid compartment (Figure S1C). At 5 weeks after MAA infection there was a striking reduction of lymphoid cells. The numbers of splenic B cells, CD4^+^ and CD8^+^ T cells were 3- to 4-fold lower when compared to spleens from uninfected mice. Reduction of CD4^+^ T cells was also observed in MAH-infected mice but to a considerably lower degree (Figure S1D).

We tested whether increased apoptosis accounted for the lymphoid ablation. However, enhanced levels of activated caspase 3 indicative for apoptosis were not detected by immunohistochemistry. Instead, apoptotic events in the spleens of infected mice appeared to decrease with time of infection (Figure S1E).

To determine whether Gr-1^+^ splenic myeloid cells contributed to pathology, we depleted Gr-1-expressing cells in MAA- and MAH-infected mice. Accordingly, anti-Gr-1 depleting antibody was administered intraperitoneally on day 11, 14, and 17 and mice were sacrificed on day 20. As seen by flow cytometry, anti-Gr-1 treatment reduced the number of Gr-1^int^CD11b^hi^CD11c^int^ cells in spleens of MAA-infected mice to the level observed in MAH-infected mice, while the numbers of other Gr-1^int^CD11b^hi^ cells were slightly increased in depleted MAA-infected mice and of Gr-1^hi^CD11b^hi^ cells were not influenced. In contrast, Gr-1 depletion did not affect the myeloid cell pool in MAH-infected mice (Figures 2D–F, Figures S2D–G). This is consistent with previous reports where anti-Gr-1 treatment results in increased granulopoiesis after initial depletion (32, 33) At necropsy, the splenomegaly induced by MAA was reduced by the anti–Gr-1 treatment. This was not observed in MAH-infected mice treated in the same manner (Figure 2G). Plating of liver homogenates from MAA- or MAH-infected Gr-1-depleted mice revealed that the anti–Gr-1 treatment reduced the bacterial burden only in MAA-infected mice. Correspondingly, histo-morphometry of HE stained liver sections revealed reduced granuloma sizes and liver pathology in MAA-infected, but not in MAH-infected mice after anti-Gr-1 treatment (Figures 2H–J, Figure S2H). Together, these results clearly demonstrate a strong impact of Gr-1^int^CD11b^hi^CD11c^int^ cells for disease aggravation in MAA-infected mice.

### Gr-1^int^CD11b^hi^CD11c^int^Ly6c^hi^ cells are monocytic myeloid derived suppressor cells and heavily infected with MAA

Next, we further characterized the Gr-1^int^CD11b^hi^ cells mediating disease aggravation. To this end, we analyzed CD11b^hi^CD11c^neg^ (P3) and CD11b^hi^CD11c^int^ (P4) cells for their Gr-1 and Ly6C expression. The CD11b^hi^CD11c^neg^ cells could be separated into Gr-1^hi^CD11b^hi^CD11c^neg^Ly6C^int^ (P6) and Gr-1^int^CD11b^hi^CD11c^neg^Ly6C^hi^ (P7) cells (Figure 3A). In addition, nearly all CD11b^hi^CD11c^int^ (P4) were found to be monocytic Gr-1^int^CD11b^hi^CD11c^int^Ly6C^hi^ cells (P5). Cytospins of these cells were ZN stained to analyze mycobacterial load. As shown in Figure 3B, MAA was almost exclusively found in the monocytic Gr-1^int^CD11b^hi^CD11c^int^Ly6C^hi^ cell population (P5), whereas the other two monocytic populations (P6 and P7) were largely ZN-negative.

To corroborate the findings of the ZN staining, DNA was extracted from the sorted cells and bacterial content was evaluated by qPCR using primers for the mycobacteria-specific insertion sequence 901 (IS901). Signals were normalized to the number of host cells by qPCR using primers for the promoter of macrophage inflammatory protein 2 (*mip2*). As expected, this analysis confirmed the almost exclusive presence of MAA DNA in the monocytic Gr-1^int^CD11b^hi^CD11c^int^Ly6C^hi^ cell population (P5; Figure 3C).

**Figure 4:**
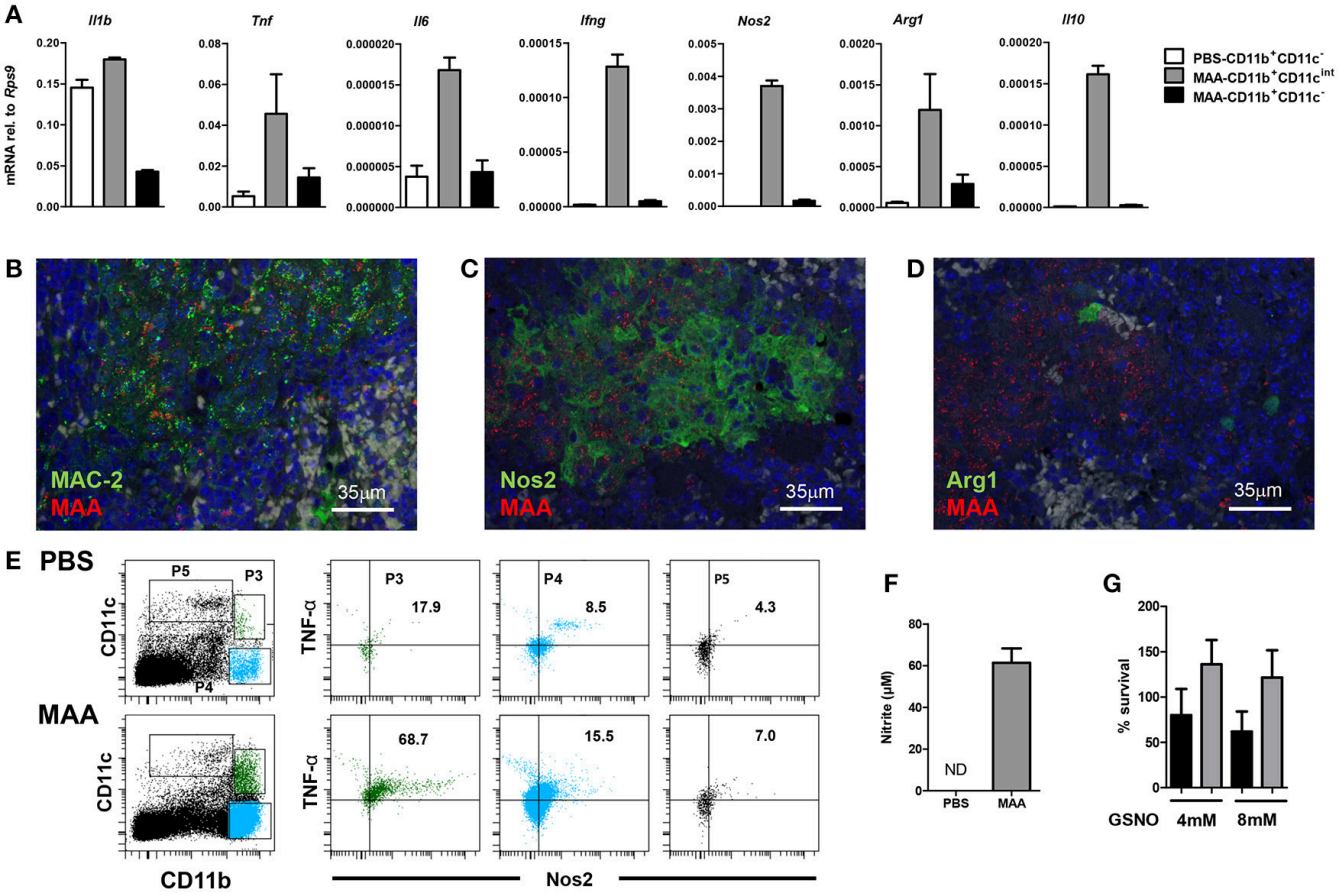
Characterization of Gr-1^int^CD11b^hi^CD11c^int^M-MDSC from MAA-infected mice. **(A)** Spleen cell suspensions from mice 5 weeks post infection were FACS sorted based on the expression level of CD11b and CD11c markers. RNA was extracted and gene expression of the indicated genes was analyzed by qRT-PCR. Graphs show the amount of mRNA relative to that of *Rps9*. **(B–D)** Fluorescence-immunohistological pictures showing splenic expression of MAC-2 **(B)**, Nos2 **(C)**, and Arg1 **(D)** (all shown in green) in mice infected for 5 weeks with MAA (red). Nuclei are stained with DAPI (blue) and tissue autofluorescence is shown in gray. **(E)** Flow cytometry of spleen suspensions from MAA-infected mice showing the percentage of Nos2 and TNFα expressing cells among different myeloid cell subpopulations. **(F)** Nitrite production of splenocytes from MAA-infected mice determined by Griess reagent. **(G)** *In vitro* NO susceptibility of MAA and MAH. Percentage of survival of MAH (black bar) and MAA (gray bar) in the presence of GSNO for 4 h. Data shown [except E (one time with 4 mice per group)] is representative of at least two experiments with 3-5 mice per group, (mean ± SEM).

Next we extracted RNA from sorted splenic CD11b^hi^CD11c^int^ and CD11b^hi^CD11c^neg^ cells (Figure 3 P4 and P3, respectively) and mRNA expression of selected genes was determined by qRT-PCR. Infected CD11b^hi^CD11c^int^ cells expressed comparatively little *Il1b* (Figure 4A). In contrast, mRNA expression of other pro-inflammatory cytokines like *Ifng, Tnf* as well as *Il6* was considerably higher than in CD11b^hi^CD11c^neg^ cells from infected and control animals. Likewise, mRNA expression of the anti-inflammatory cytokine *Il10* as well as of *Arg1* and *Nos2* were markedly higher in CD11b^hi^CD11c^int^ cells as compared to uninfected control mice or CD11b^hi^CD11c^neg^ cells from MAA-infected animals (Figure 4A).

The mRNA expression profile of infected cells was confirmed by immuno-histochemistry. Tissue sections of spleens of MAA-infected mice were stained with antibodies against the common monocyte/macrophage marker Mac2 (galectin 3) (34) and the mycobacterial hemagglutinin binding protein HBHA (35). As shown in Figure 4, MAA was associated with Mac2 expressing cells. In addition, mycobacteria-positive areas could be stained with antibodies against Nos2 or Arg1, indicating that myeloid cells in such areas expressed Nos2 and some of them also Arg-1 (Figures 4B–D). Furthermore, strong intracellular Nos2 and TNF expression was observed for CD11b^hi^CD11c^int^ cells by flow cytometry (Figure 4E). Finally, medium conditioned by splenocytes from MAA-infected mice contained large amounts of nitrite as stabile metabolite of NO as detected by Griess reaction (Figure 4F). Overall, the immature phenotype of the MAA-infected monocytic Gr-1^int^CD11b^hi^CD11c^int^Ly6C^hi^ cell population as well as the expression of TNF, Nos2, and Arg1 strongly suggested that these cells represent a monocytic myeloid-derived suppressor cell (M-MDSC) population.

The residence of MAA in these NO-producing cells and the proliferation in an NO/nitrite-containing milieu of the spleen suggested resistance of the MAA strain against NO. Indeed, treatment of MAA and MAH with the NO donor GSNO (4 and 8 mM) for 4 h did not influence viability of MAA although it considerably affected MAH (Figure 4G).

### NO produced by Gr-1^int^CD11b^hi^CD11c^int^ M-MDSC is responsible for CD4^+^ T cell ablation and disease control

**Figure 5:**
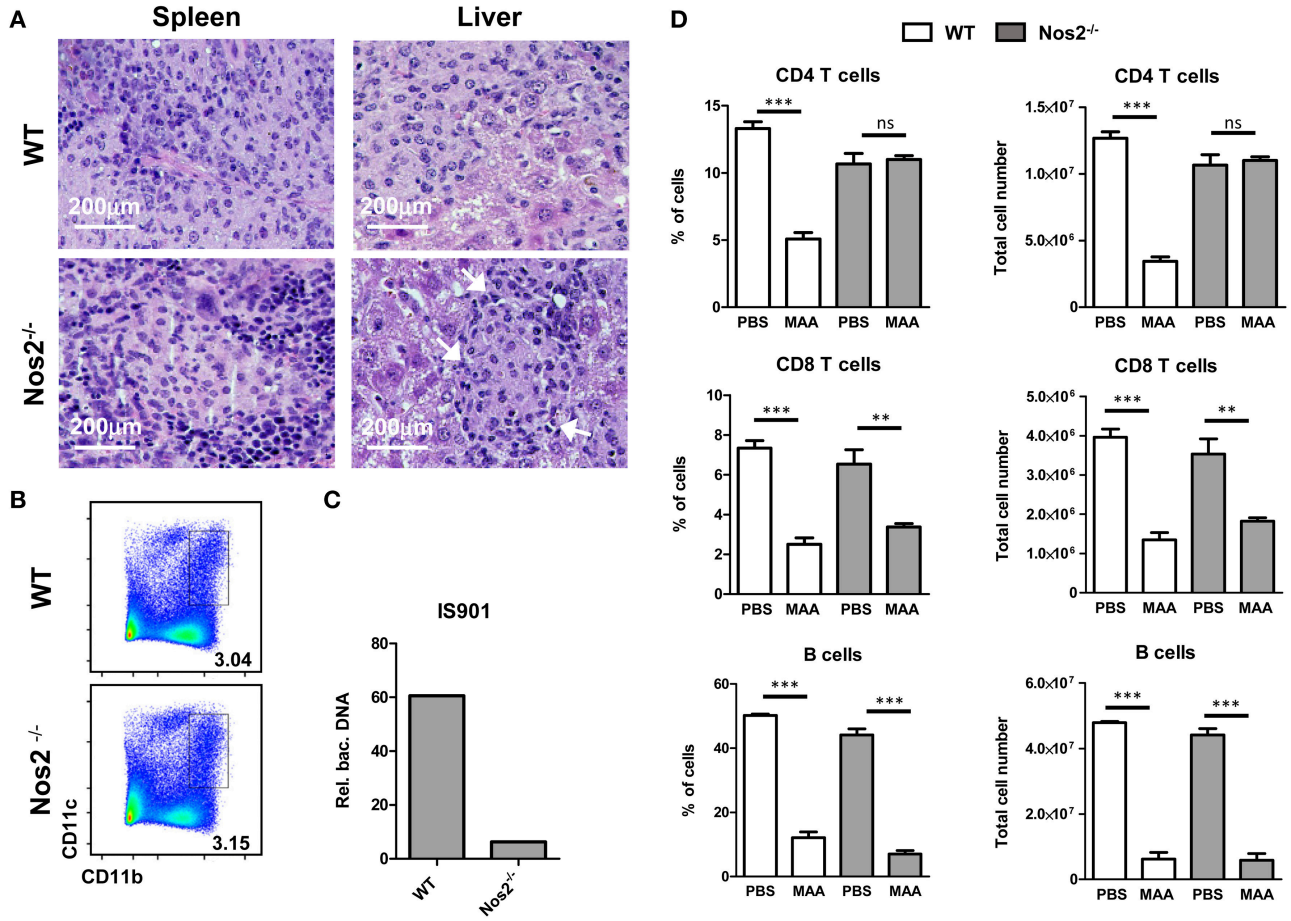
NO produced by Gr-1^int^CD11b^hi^CD11c^int^ M-MDSC is responsible for CD4^+^ T cell ablation and disease control. **(A)** HE stained histology of spleen and liver from MAA-infected wt and Nos2^−/−^ mice. Arrows indicate areas of lymphocytic cells. **(B)** FACS plots of spleen suspensions showing the percentage of M-MDSC like cells from MAA-infected wt and Nos2^−/−^ mice. **(C)** Amount bacterial DNA in M-MDSC from MAA-infected wt and Nos2^−/−^ mice determined as described in (Figure 3C). **(D)** Graphs showing the percentage (right) and the total number (left) of lymphocytes of spleens from MAA-infected wt and Nos2^−/−^ mice 5 weeks pi. Data from 4 mice per group are shown. ^**^p < 0.005, ^***^p < 0.001, *t*-test.

NO can exert a direct pro-apoptotic and/or anti-inflammatory effect in T cells (19, 36). Even though apoptotic events were low at the tissue level, the question arose whether ablation of T and B cells in the spleen of MAA-infected mice is due to production of NO by Gr-1^int^CD11b^hi^CD11c^int^ M-MDSC. Therefore, we infected Nos2^−/−^ mice with MAA. After 5 weeks, spleens of MAA-infected wild-type (wt) and Nos2^−/−^ mice showed a massive influx of histiocytic cells. In contrast to wt mice, MAA-infected Nos2^−/−^ mice exhibited mostly granuloma harboring more outer rim lymphocytes (Figure 5A).

Interestingly, spleens of MAA-infected Nos2^−/−^ mice exhibited similar frequency of M-MDSC-like Gr-1^int^CD11b^hi^CD11c^int^ cells as MAA-infected wt mice (Figure 5B). However, in contrast to wt Gr-1^int^CD11b^hi^CD11c^int^ cells, Nos2^−/−^ Gr-1^int^CD11b^hi^CD11c^int^ cells harbored lower amounts of bacteria (Figure 5C). Ablation of B cells and CD8^+^ T cells remained unaltered in Nos2^−/−^ mice, whereas the CD4^+^ T cell compartment was fully restored (Figure 5D). Thus, in chronically MAA-infected mice NO produced by Gr-1^int^CD11b^hi^CD11c^int^ M-MDSC appears to exacerbate the infection and affects specifically CD4^+^ T cells.

### NO produced by Gr1^int^CD11b^hi^CD11c^int^ M-MDSC affects CD4^+^ T cell responses and cDC function *ex vivo*

**Figure 6:**
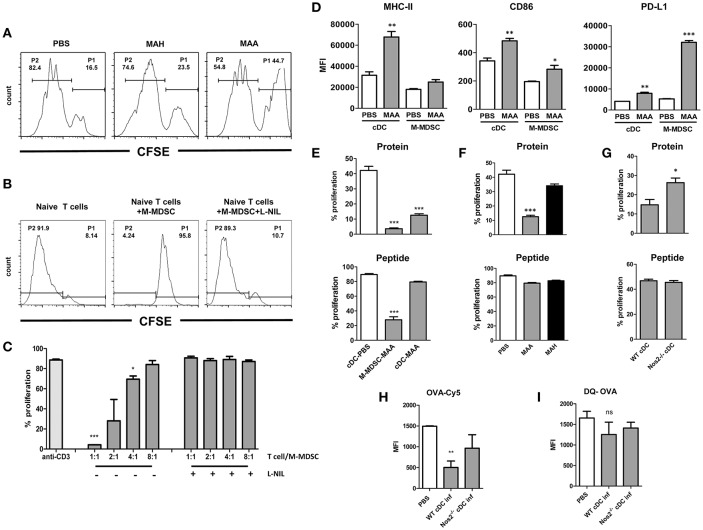
NO produced by Gr-1^int^CD11b^hi^CD11c^int^M-MDSC from MAA-infected mice affects CD4^+^ T cell response and cDC function *ex vivo*. **(A)** Anti-CD3 antibody mediated spleen CD4^+^ T proliferation from naïve (PBS control) and from mice infected with MAH or MAA for 33 days. CD4^+^ T cells from 5 mice in each group were isolated, pooled, CFSE labeled and cultured on anti-CD3 antibody coated cell culture microplates for 4 days. Histograms showing the percentage of non-proliferating cells (P1) and proliferating cells (P2). **(B)** CD11b^hi^CD11c^int^ cells from spleens of MAA-infected mice were FACS sorted and co-cultured with CFSE labeled naïve CD4^+^ T cells in the presence of plate bound anti-CD3 antibody. T cell proliferation was measured after 4 days in culture by determining CFSE dilution. **(C)** Graph shows percentage of proliferation after incubating 3 × 10^5^ CD4^+^ T cells with decreasing numbers of M-MDSC in the presence or absence of Nos2 inhibitor, L-N6-(1-iminoethyl) lysine di-hydrochloride (L-NIL) at a final concentration of 40 μM. Data shows two independent experiments (mean ± SEM, ^*^p < 0.05, ^***^p < 0.001). **(D)** Expression level of MHC-II, CD86, PD-L1 of conventional dendritic cells (cDC) and CD11b^hi^CD11c^int^cells (M-MDSC) from MAA-infected and PBS control mice determined by flow cytometry. MFI – mean fluorescent intensity (mean ± SEM, ^*^p < 0.05, ^**^p < 0.01, ^***^p < 0.001) from two independent experiments with 3-5 mice per group are shown. **(E)** M-MDSC and cDC from MAA-infected and control mice (PBS) were sorted from spleen suspensions and pulsed with ovalbumin protein (100 μg/ml) or ovalbumin peptide 323-339 (1 μg/ml) for 1 h at 37°C. Subsequently, they were incubated with CFSE labeled splenic CD4^+^ T cells isolated from OT-II mice. After 3 to 4 days, proliferation of T cells was measured by flow cytometry. Graphs show the percentage of T cell proliferation stimulated by OVA protein (top) or OVA peptide (bottom). **(F)** Percentage of T cell proliferation induced by cDC from MAA or MAH-infected mice or control mice (PBS) analyzed as described in **(E)**. **(G)** T cell proliferation induced by cDC from MAA-infected wt and Nos2^−/−^ mice analyzed as described in **(E)**. **(H,I)** Uptake and processing of protein antigen by cDC. **(H)** cDC from control and infected mice were exposed to Cy5 conjugated OVA for 1.5 h and the amount of internalized antigen was determined by flow cytometry. **(I)** Degradation of internalized was determined using DQ OVA. As fluorescence is quenched on intact DQ-OVA, only degraded OVA can be determined by flow cytometry. Results from 3-5 mice were included per group and three independent experiments performed (mean ± SEM, ^*^p < 0.05, ^***^p < 0.001).

NO derived from M-MDSC or other myeloid cells is capable to directly suppress T cells activation (19, 36). To see whether this phenomenon is also seen in MAA- or MAH-infected mice, we isolated splenic CD4^+^ T cells and stimulated them with anti-CD3 or anti-CD3 plus anti-CD28. As shown in Figure 6A, CD4^+^ T cells from MAA-infected mice exhibited significantly reduced proliferation compared to uninfected control mice or CD4^+^ T cells from MAH-infected mice. Next, we tested whether the suppressive activity of splenic Gr-1^int^CD11b^hi^CD11c^int^ M-MDSC from MAA-infected mice is dependent on Nos2-derived NO. Isolated control (naïve) CD4^+^ T cells were stimulated with anti-CD3 in the presence or absence of splenic Gr-1^int^CD11b^hi^CD11c^int^ cells from MAA-infected mice. Strong proliferation was observed in the absence of Gr-1^int^CD11b^hi^CD11c^int^ M-MDSC (Figure 6B). In contrast, when Gr-1^int^CD11b^hi^CD11c^int^ M-MDSC from MAA-infected mice were present, proliferation was significantly lower. Inhibition correlated with the ratio of M-MDSC to T cells (Figure 6C). Importantly, the inhibitory activity of Gr-1^int^CD11b^hi^CD11c^int^ M-MDSC was completely abolished, when the Nos2 inhibitor L-N^6^-(1-iminoethyl) lysine di-hydrochloride (L-NIL) was added to the co-cultures (Figures 6B,C). These data suggest that Gr-1^int^CD11b^hi^CD11c^int^ M-MDSC from MAA-infected mice exhibit Nos2/NO-dependent inhibitory activity toward T cells *ex vivo*.

While NO can directly inhibit CD4^+^ T cell proliferation, NO is also known to modulate the function of antigen-presenting cells (20, 21, 37). Thus, we analyzed the dominant cell populations expressing CD11c, i.e. conventional dendritic cells (cDC; CD11c^high^CD11b^+/−^) as well as Gr-1^int^CD11b^hi^CD11c^int^ M-MDSC for surface markers involved in the activation or inhibition of T cells. MHC class II and costimulatory CD86 were found on cDC and Gr-1^int^CD11b^hi^CD11c^int^ M-MDSC from PBS-treated control mice and were upregulated upon MAA infection (Figure 6D). Interestingly, expression of programmed death ligand 1 (PD-L1) was significantly higher on Gr-1^int^CD11b^hi^CD11c^int^ M-MDSC of MAA-infected mice as compared to cDC, which would be compatible with direct involvement of Gr-1^int^CD11b^hi^CD11c^int^ M-MDSC in negative T cell regulation.

For functional testing, we first investigated T cell stimulatory capacity of cDC and Gr-1^int^CD11b^hi^CD11c^int^ M-MDSC. Isolated cDC (>95% purity) from MAA-infected or control mice were sensitized with ovalbumin (OVA) and co-incubated with OVA specific OTII CD4^+^ T cells. Strong proliferation was observed with cDC from uninfected, PBS-treated mice, whereas cDC from MAA-infected mice were strikingly impaired in their T cell stimulatory capacity (Figure 6E, top). This could indicate a defect in the antigen-processing ability as OVA protein requires processing. Therefore, the experiment was repeated by using an OVA-derived peptide as antigen which does not require processing. Strong OTII T cell proliferation was elicited by both cDC populations under these conditions (Figure 6E, bottom). Apparently, antigen-presentation and T cell stimulation is undisturbed in cDC from MAA-infected mice, but antigen-processing might be altered. This was in agreement with the sustained expression of MHC-II and the CD86 co-stimulatory molecules. In contrast, Gr-1^int^CD11b^hi^CD11c^int^M-MDSC had considerably lower capacity to directly stimulate OT-II T cells with either protein or peptide (Figure 6E).

It is possible that splenic cDC from MAA-infected mice could be directly infected by mycobacteria. This might affect their antigen-processing compartments as has already been suggested for infected macrophages (38). However, cytospins of sorted cDC from MAA-infected mice revealed that hardly any cDC were positive for mycobacteria (Figure S3A).

We then studied whether impaired antigen-processing is a general feature of mycobacterial infection or specific for cDC from MAA-infected mice. Splenic cDC were sorted from MAH- or MAA-infected mice and assessed for their capacity to process and present antigen. As shown in (Figure 6F, Figure S3B), impairment of cDC sensitized with OVA protein was specific for MAA-infected mice, while cDC from MAH-infected mice were fully capable to induce CD4^+^ T cell proliferation. The T cell stimulatory capacity was retained when OVA peptide was used as seen before (Figures 6E,F, bottom). Inhibition of antigen-processing required viable mycobacteria since administration of heat-killed MAA did not result in suppression of T cell stimulation (Figure S3C).

The impaired antigen-processing capacity of cDC from MAA-infected mice might be due to NO produced by the M-MDSC. Therefore, splenic cDC were sorted from MAA-infected wt or Nos2^−/−^ mice and assessed for their capacity to process and present antigen. As shown in Figure 6G, cDC from infected Nos2^−/−^ mice induced significantly higher OTII T-cell proliferation than cDC from infected wt mice.

Next, we analyzed whether the antigen uptake or processing was affected in cDC from MAA-infected wt mice. As shown in Figures 6H,I, OVA-Cy5 uptake of cDC from infected wt mice was severely impaired, while it was improved in cDC from infected Nos2^−/−^ mice. Surprisingly, cDC from all groups of mice had comparable levels of DQ-OVA signals, which is an indicator for intact antigen-processing. To localize the trafficking of DQ-OVA in the cellular compartments, cDC from wt and Nos2^−/−^ mice were stained with LAMP-1, an indicator of late endosome. As shown in Figure S4, co-localization of DQ-OVA and LAMP-1 was observed in both groups. Since cDC from MAA-infected wt mice apparently took up antigen less efficiently, there must be a compensatory enhancement of antigen processing in such cells.

Overall, these data provide clear evidence that in the spleens of MAA-infected mice NO derived from M-MDSC is also responsible for a defect in the T cell stimulatory capacity of cDC. Nevertheless we cannot exclude the possibility that other NO producing cells might be involved.

### Occurrence of Gr-1^int^CD11b^hi^CD11c^int^M-MDSC correlates with T cell inhibition in MAA-infected mice

**Figure 7:**
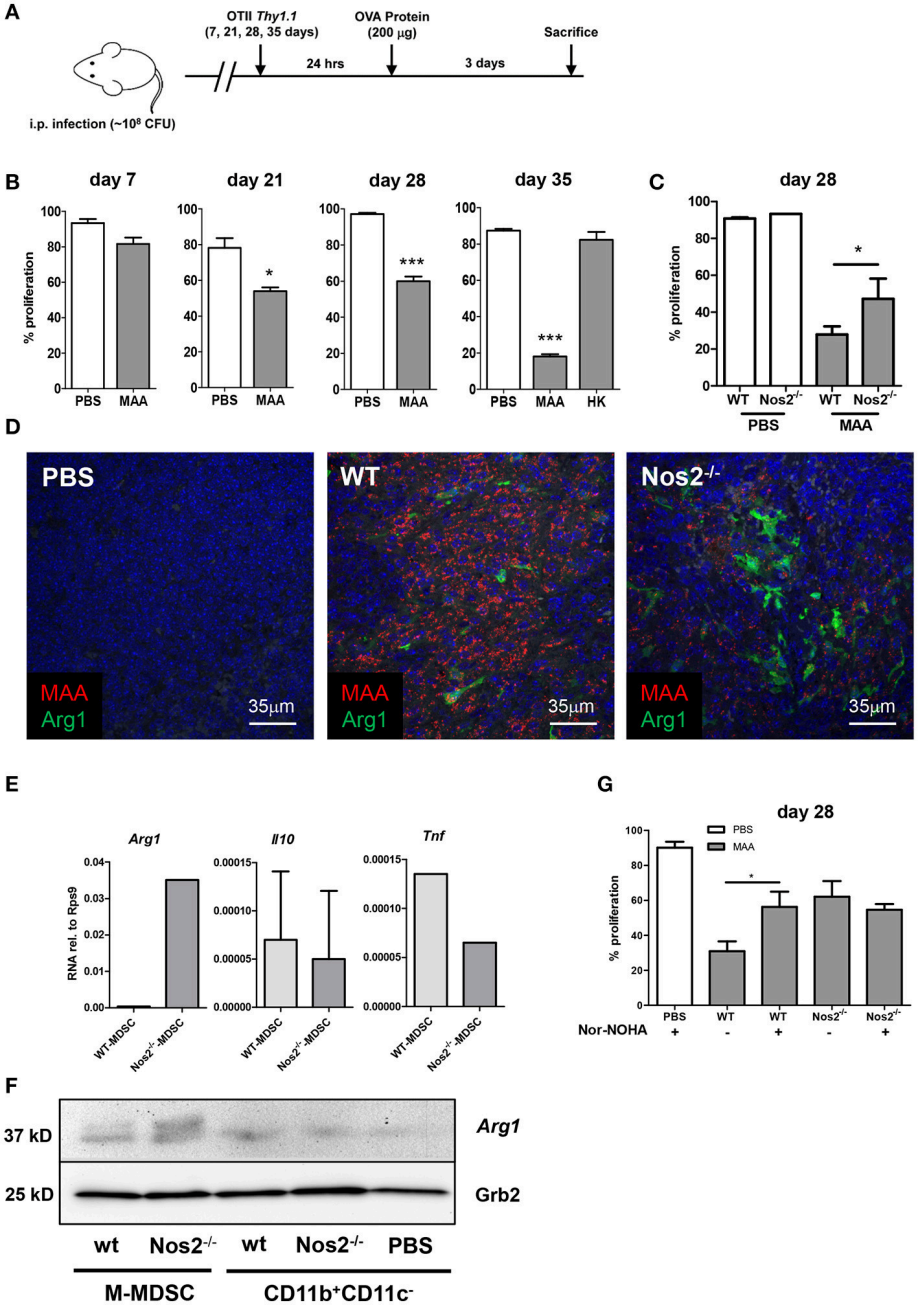
Occurrence of Gr-1^int^CD11b^hi^CD11c^int^M-MDSC correlates with T cell inhibition in MAA-infected mice. **(A)** Experimental scheme for *in vivo* T cell proliferation analyses. **(B)** Percentage of *in vivo* T cell proliferation at the indicated time points. Mice were infected with MAA or treated with heat inactivated MAA (HK). At the indicated time point after infection 2 × 10^6^ CFSE labeled OT-II CD4^+^ T cells were transferred intravenously. After 24 h, 200 μg ovalbumin protein was injected i.p. Three days after immunization, mice were sacrificed and spleen suspensions were analyzed for proliferation of OT-II CD4^+^ T cells by flow cytometry (Mean ± SEM, ^*^p < 0.05, ^***^p < 0.001, *t*-test). **(C)** *In vivo* CD4^+^ T cells proliferation in MAA-infected WT and Nos2^−/−^ 28 days post infection. Analyses were performed as described in **(A)** (mean ± SEM, ^*^p < 0.05, *t*-test). **(D)** Fluorescence immunohistochemistry of Arg1 (green) expression in spleen of MAA (red) infected wt mice and Nos2^−/−^ mice. Nuclei are stained with DAPI (blue) and tissue autofluorescence is shown in gray. **(E)** Splenic M-MDSC were sorted from wt and Nos2^−/−^ mice infected for 28 days with MAA. RNA was extracted and qRT-PCR was performed. Results were displayed as described in Figure 3A. Results from 3-4 mice per group (mean ± SEM). **(F)** Protein expression level of Arg1 among different cell populations isolated from spleen as analyzed by immunoblotting. Spleen myeloid cell populations were sorted 35 days post infection from MAA-infected wt and Nos2^−/−^ mice [wt Gr-1^int^CD11b^hi^CD11c^int^M-MDSC; Nos2^−/−^ Gr-1^int^CD11b^hi^CD11c^int^M-MDSC; wt CD11b^+^CD11c^−^ cells; Nos2^−/−^ CD11b^+^CD11c^−^ cells; CD11b^+^CD11c^−^ cells from uninfected mice (PBS)]. **(G)** Wt and Nos2^−/−^ mice infected for 3 weeks by MAA and uninfected controls (PBS) were treated with i.p. injection of nor-NOHA (2 mg/mouse/day) 2 days prior to CD4^+^ T cell transfer and for 3 consecutive days after T cell transfer. Percent of *in vivo* proliferating CD4^+^ T cell is displayed. Results show two independent experiments and 3-5 mice were included per group (mean ± SEM, ^*^p < 0.05, *t*-test).

The above data clearly demonstrated that splenic Gr-1^int^CD11b^hi^CD11c^int^M-MDSC as well as cDC from MAA-infected mice when analyzed *ex vivo* hindered CD4^+^ T cell proliferation. These effects were Nos2/NO dependent. As the *in vivo* situation is significantly more complex, we also analyzed T cell proliferation in MAA-infected mice. To this end, mice were infected with MAA. Then, OVA-specific CD4^+^ OT-II T cells labeled with CFSE were adoptively transferred at 1, 3, 4, and 5 weeks p.i. according to the schedule displayed in Figure 7A. After 24 h, antigen-specific *in vivo* proliferation was induced by i. p. administration of OVA. Three days later, proliferation of CD4^+^ OT-II T cells was evaluated. Consistent with the histology shown before (Figure 1D), *in vivo* CD4^+^ T cell proliferation was not affected during the initial phase of infection. However, after 3 weeks an inhibitory effect on proliferation was first observed. By 5 weeks of infection, T cell proliferation was strongly reduced. This inhibition was only seen when viable bacteria were applied (Figure 7B). Thus, T cell-inhibitory activity positively correlated with severity of inflammation and with the appearance of Gr-1^int^CD11b^hi^CD11c^int^M-MDSC in the spleen of MAA infected mice (Figure 1).

To investigate, whether *in vivo* inhibition of T cell proliferation was dependent on NO as observed *ex vivo*, we tested OTII CD4^+^ proliferation using MAA-infected Nos2^−/−^ mice. Similar to the *ex vivo* results in Figure 6, CD4^+^ OT-II T cell proliferation was significantly improved in Nos2^−/−^ mice infected with MAA. However, proliferation did not reach the levels observed in uninfected mice (Figure 7C). This suggested that even though NO production by Gr-1^int^CD11b^hi^CD11c^int^M-MDSC was abolished in Nos2^−/−^ mice, alternative T cell-inhibitory mechanisms were still active. As the population of Gr-1^int^CD11b^hi^CD11c^int^M-MDSC was numerically unaltered in MAA-infected Nos2^−/−^ mice, we assumed that M-MDSC inhibited T cell proliferation under *in vivo* conditions via compensatory mechanisms such as the expression of Arg1 which we had already seen in M-MDSC from MAA-infected mice (Figure 4A). Indeed, higher Arg1 protein expression was observed in splenic sections of MAA-infected mice in the absence of Nos2 (Figure 7D). This was confirmed at cellular level. Gr-1^int^CD11b^hi^CD11c^int^M-MDSC sorted from MAA-infected mice lacking Nos2 expressed roughly 100-fold higher mRNA levels of *Arg1* than Gr-1^int^CD11b^hi^CD11c^int^ M-MDSC from infected wt mice. Expression of mRNA encoding *Tnf* was reduced and *Il10* was nearly unaffected (Figure 7E). Upregulation of Arg1 protein in Gr-1^int^CD11b^hi^CD11c^int^ M-MDSC in absence of Nos2 was confirmed by immunoblot (Figure 7F). Thus, in Nos2^−/−^ mice the lack of Nos2 i.e. NO appeared to be functionally compensated by Arg1.

Arg1 expressed by M-MDSC is known to cause T cell inhibition by depletion of the semi-essential amino acid L-arginine (19, 39). Therefore, to evaluate the effect of Arg1 expression, we treated MAA-infected wt mice or Nos2^−/−^ mice with the arginase inhibitor N^ω^-hydroxy-nor-L-arginine (nor-NOHA) 2 days before and after OTII cell transfer and immunization for consecutive 6 days. As shown in Figure 7G, this treatment partially restored T cell proliferation in wt mice indicating that *in vivo* Gr-1^int^CD11b^hi^CD11c^int^ M-MDSC also impede T cell activation via Arg1 expression. Unexpectedly the treatment did not alter T cell activation in Nos2^−/−^ mice (Figure 7G) suggesting a markedly higher Arg1 activity in such mice.

## Discussion

In the present study, we unraveled the functional role of a M-MDSC population that is particularly induced after infection of mice with a highly virulent strain of *M. avium* subsp. *avium*. This cell population exhibited a hitherto unknown CD11c^int^ phenotype and considerably expanded during the progression of the disease. M-MDSC acted as target cells for mycobacterial growth, created an inflammatory milieu prone to cause T cell suppression and aggravated the infection by the expression of Nos2 and Arg1.

The interaction of CD4^+^ T cells with antigen-presenting cells plays a critical role in controlling mycobacterial disease (40, 41). Studies on bacterial survival and CD4^+^ T cell responses after infection of mice with *M. avium* revealed that the result of infections and immunopathology strongly depended on the subspecies and strain of *M. avium* investigated and the route of infection (12, 42–44). Therefore, to dissect the relevance of myeloid cells during chronic *M. avium* infection of mice, we used the MAA strain 25291, commonly used as a highly virulent model organism for disseminated mycobacterial disease (45), and the MAH strain 104, which shows intermediate virulence (11, 46). We selected the intraperitoneal route of infection as it results in fast systemic dissemination and reproducible chronic infection with granulomatous inflammation in deep organs.

Five weeks after inoculation, mice exhibited systemic mycobacterial infection regardless of the strain used. As expected, more severe disease and higher bacterial burden were observed in MAA-infected mice. However, granulomatous inflammation in liver and spleen was induced by both strains as described before (11, 12, 47). Importantly, the quality of the granulomatous inflammation in both liver and spleen considerably differed between mice infected with MAA or MAH. The granulomatous inflammation in MAA-infected mice was particularly characterized by the appearance of increasing numbers of mononuclear cells. The high production of IL-6 and CCL5 in MAA-infected mice is most likely responsible for this accumulation. Both cytokines have been shown to induce proliferation of MDSC in tumors (48, 49).

Phenotypic characterization of the splenic monocytic population in MAA- and MAH-infected mice revealed that the overall number of Gr-1 and CD11b expressing cells increased. However, accumulation of Gr-1^int^ cells in MAA-infected mice was significantly higher. Cells expressing Gr-1 and CD11b markers have been described as MDSC (50). Such cells are classified as polymorphonuclear or PMN-MDSC, when expressing CD11b^+^Ly6G^+^Ly6C^lo^, and as M-MDSC, when expressing CD11b^+^Ly6G^−^Ly6C^hi^ (51). M-MDSC usually lack surface markers of monocytes such as CD11c and MHC class II (15). In contrast, Gr-1^int^ cells in MAA-infected mice exhibited a Gr-1^int^CD11b^hi^CD11c^int^ phenotype, expressed high levels of Ly6C as well as MHCII and CD86. Nevertheless, these cells were unable to present antigen to CD4^+^ T cells but fulfilled criteria of M-MDSC, both functionally and phenotypically (14). They expressed PD-L1, IL-10, Arg1 and Nos2, produced NO and inhibited T cell activation.

The immune-regulatory activity of M-MDSC on T cells largely depends on the metabolic consumption of arginine by the activity of the inducible enzymes Nos2 or Arg1 (14, 17). M-MDSC thereby create an arginine starved milieu which prevents T cell growth (52). In addition, NO suppresses T cell function through various mechanisms, including the alteration of signaling cascades in T cells, the inhibition of MHC class II expression, and the induction T cell apoptosis (17, 19, 20). Indeed, immunohistochemistry revealed that in MAA-infected mice splenic M-MDSC harboring mycobacteria expressed high levels of Nos2 and moderate levels of Arg1 protein. Furthermore, we were able to show that the ablation of CD4^+^ T cells observed in the spleens of MAA-infected mice was restored in Nos2^−/−^ mice. This pointed toward an Nos2/NO-mediated immunosuppressive activity of the Gr-1^int^CD11b^hi^CD11c^int^ M-MDSC. In agreement, our *ex vivo* analyses of Gr-1^int^CD11b^hi^CD11c^int^ M-MDSC from MAA-infected mice showed that these cells were able to inhibit T cell proliferation in a Nos2/NO-dependent manner.

During mycobacterial infection, DC execute two key functions: participating in antigen presentation and in granuloma formation (53). In the present study we showed that the suppressive activity of Gr-1^int^CD11b^hi^CD11c^int^ M-MDSC was not only limited to T cells but also affected the splenic DC population. Absence of T cell stimulatory capacity seems to be specific for DC from MAA-infected mice and partially restored in Nos2^−/−^ mice. Obviously, NO from M-MDSC influenced the up-take and/or processing of protein antigen, but not its presentation, since the presentation of peptide remained intact. Our findings are reminiscent to the findings of Zietara et al. in RAG deficient mice (30). They are a novel example to an emerging number of reports demonstrating a direct or indirect influence of MDSC on other immune cells including DC. For instance, MDSC have been reported to impair DC functions in mouse tumor models and thereby aggravated tumor-induced immune suppression and tumor growth (54–56). However, our data, for the first time demonstrate a critical role of NO produced by M-MDSC in these processes.

In addition to DC, CD4^+^ T cells of MAA-infected mice were impaired as well. Splenic T cells purified from MAA-infected mice were not responsive to CD3/CD28 stimulation. They could not be stimulated *ex vivo*. *Ex vivo* data suggested that the lack of T cell response was due to NO production by Gr-1^int^CD11b^hi^CD11c^int^ M-MDSC. To confirm these findings in the more complex *in vivo* situation, we took advantage of adoptive transfer of ovalbumin-specific OT II cells. These experiments clearly demonstrated that during late phases of MAA infection antigen-specific proliferation of CD4^+^ T cells was inhibited. The extent of inhibition of OT II cell proliferation correlated with the extent of pathology in the spleens and the number of mycobacteria-containing cells. The inhibition of T cells in the spleen appeared to be of general nature and not restricted to MAA-specific CD4^+^ T cells.

Interestingly, Gr-1^int^CD11b^hi^CD11c^int^ M-MDSC were even induced in the absence of Nos2/NO as shown using Nos2^−/−^ mice. This is in line with studies on MTB infected Nos2^−/−^ mice (29). However, it contrasts with studies on tumors where Nos2/NO were shown to be essential for the induction of MDSC (57).

The deletion of Nos2 had a positive effect on the overall number of CD4^+^ T cells emphasizing the detrimental effect of Nos2/NO on the proliferation of these cells. However, OT II T cell proliferation was only partially restored in Nos2^−/−^ mice, indicating that Gr-1^int^CD11b^hi^CD11c^int^ M-MDSC are able to express an alternative mechanism of suppression. We showed that in the absence of Nos2/NO, Arg1 influenced OT II cell proliferation in MAA-infected mice. Indeed, Arg1 was highly expressed under these conditions. It is known that the products of Arg1 and Nos2 reciprocally regulate each other's enzymatic activity (58). Thus, polyamines produced by the Arg1/ornithine decarboxylase pathway are capable to inhibit Nos2 activity, whereas N-hydroxy-L-arginine (LOHA), the intermediate of the Nos2 reaction, functions as an inhibitor of arginase (58). Furthermore, inhibitors of Nos2 transcription have been shown to upregulate Arg1 expression in murine macrophages (59). In our study, we observed that in the absence of Nos2 the expression of TNF mRNA was reduced. As TNF is a potent negative regulator of the expression of Arg1 (22), the deficiency of TNF might have contributed to the enhanced expression of Arg1 seen in Nos2^−/−^ mice.

In line with previous studies (60), our data confirm that the absence of Nos2 in MAA-infections improved bacterial clearance. This is in remarkable contrast to MTB where infected mice lacking Nos2 suffer from severe TB disease (61–63) confirming striking differences in the pathogenicity of virulent mycobacteria (60). The higher resistance of Nos2^−/−^ mice might result from (a) the normalized number and function of CD4^+^ T cells or (b) the absence of NO-mediated detrimental effects on other immune cells such as DC. Nevertheless, formation of classical granuloma during MAA-infection might be sufficient to reduce bacterial spreading even in the presence of T cell suppressive M-MDSC activity.

In conclusion, the results of this study demonstrated the critical role of M-MDSC in mycobacterial infection. By *ex vivo* and *in vivo* analyses we showed that the ability of NTM to cause accumulation of M-MDSC with a unique Gr-1^int^CD11b^hi^CD11c^int^ phenotype is an important virulence trait. The monocytic nature of such M-MDSC rendered them susceptible to mycobacterial replication. Their immature phenotype and their ambiguous response to infection considerably influenced the outcome of the local innate and adaptive immune response, thereby facilitating progression of mycobacterial disease. In addition, our study provided evidence that M-MDSC utilize distinct mechanisms like expression of Nos2 and Arg1 to suppress T cells during mycobacterial infection, either directly or indirectly via a Nos2/NO-dependent impairment of the function of DC.

## Author contributions

KA, SW, and RG designed experiments. KA, AN, NJ, NR, UH, VP, and CF performed the experiments. KA, AB, US, CB, DB, SW, and RG analyzed the data. CB provided methodological advice and contributed to the writing of the manuscript. KA, SW, and RG wrote the paper.

### Conflict of interest statement

The authors declare that the research was conducted in the absence of any commercial or financial relationships that could be construed as a potential conflict of interest.

## Supplementary material

Figure S1 Related to Figures 1, 2: Chronic MAA infection induces accumulation of mycobacteria harboring histiocytic cells in murine spleen. **(A)** Patterns of serum cytokines and chemokines 5 weeks after infection as determined by multiplex ELISA. Statistics shown represent difference between MAA and MAH infected mice serum cytokines (6 mice were included for the two infected groups and 3 mice in PBS control). **(B)** Kinetics of MAA proliferation in the liver determined by plating and CFU counting. **(C)** Numbers of splenic lymphocytes of the indicated cell populations. **(D)** Flow cytometry of spleen suspensions from MAA or MAH-infected mice showing the percentage of lymphocytes in spleens 5 weeks after infection as determined by flow cytometry. **(E)** Immuno-histological pictures showing expression of cleaved caspase 3 in spleens of uninfected mice and mice infected with MAA for 7, 21, 28, and 35 days. Caspase 3 positive cells were stained brownish color (Mean ± SEM, ^*^*p* < 0.05, ^**^*p* < 0.001, ^***^*p* < 0.0001: One way ANOVA).

Figure S2 Related to Figure 2: Chronic MAA infection induces accumulation of disease-aggravating Gr-1^int^CD11b^hi^CD11c^int^ cells in the spleen. **(A–F)** Total number of the indicated cell populations/spleen. Total live spleen cells were counted using hemocytometer after adding trypan blue to exclude dead cells. Total number of cell populations/spleen in the respective graphs was calculated using the percentage of cell populations among live cells as determined by flow cytometry. **(G)** Representative flow cytometry showing anti-Gr-1 antibody mediated depletion of CD11b^high^CD11c^int^ cells which are mostly Gr-1^int^ phenotype. **(H)** Representative liver HE staining revealed the granuloma morphotype with or without anti-Gr-1 antibody treatment. The results represent at least two independent experiments (Mean ± SEM, **(A–C)** ^*^*p* < 0.05, ^**^*p* < 0.01, ^***^*p* < 0.0001, **(D–F)** *t*-test, ^*^*p* < 0.05, ^**^*p* < 0.01: One way ANOVA).

Figure S3 Related to Figure 6: NO produced by Gr-1^int^CD11b^hi^CD11c^int^M-MDSC from MAA-infected mice affected CD4 T cell and cDC function *ex vivo*. **(A)** ZN stained cDC from MAA-infected mice after 5 weeks. **(B)** Percentage of proliferated CD4^+^ T cells upon OVA protein (upper panel) and OVA peptide (Aa 323-339) (lower panel) stimulation. **(C)** Percentage of proliferated CD4^+^ T cells upon OVA protein stimulation. The result indicates only live MAA influence cDC function up on OVA protein stimulation. Result shown are representative of two independent experiments (Mean ± SEM, ^*^*p* < 0.05, ^***^*p* < 0.0001: One way ANOVA).

Figure S4 Related to Figure 6: NO produced by Gr-1^int^CD11b^hi^CD11c^int^M-MDSC from MAA-infected mice affected CD4^+^ T cell and cDC function *ex vivo*. Image showing co-localization of DQ-OVA (shown in green) and LAMP-1 (shown in red) in cDC from wt and Nos2^−/−^ mice 5 weeks post MAA infection.

Figure S5 Flow cytometry gating strategy. **(A)** FACS analysis gating strategy to analyze spleen lymphoid and myeloid cells. **(B)** Gating strategy used to determine *in vivo* CD4^+^ T cells proliferation.

Table S1 List of antibodies for flow cytometry used in this study.

## References

[1] Hoefsloot W van Ingen J Andrejak C Angeby K Bauriaud R Bemer P. The geographic diversity of nontuberculous mycobacteria isolated from pulmonary samples: an NTM-NET collaborative study. Eur Respir J. (2013) 42:1604–13. 10.1183/09031936.00149212 23598956

[2] Daley CL. *Mycobacterium avium* complex disease. Microbiol Spectr. (2017) 5. 10.1128/microbiolspec.TNMI7-0045-2017 28429679 PMC11687487

[3] Martinez Gonzalez S Cano Cortes A Sota Yoldi LA Garcia Garcia JM Alba Alvarez LM Palacios Gutierrez JJ Non-tuberculous mycobacteria. an emerging threat? Arch Bronconeumol. (2017) 53:554–60. 10.1016/j.arbr.2017.08.004 28433210

[4] Diel R Jacob J Lampenius N Loebinger M Nienhaus A Rabe KF. Burden of non-tuberculous mycobacterial pulmonary disease in Germany. Eur Respir J. (2017) 49:1602109. 10.1183/13993003.02109-2016 28446559

[5] Stout JE Koh WJ Yew WW. Update on pulmonary disease due to non-tuberculous mycobacteria. Int J Infect Dis. (2016) 45:123–34. 10.1016/j.ijid.2016.03.006 26976549

[6] Rindi L Garzelli C. Genetic diversity and phylogeny of *Mycobacterium avium*. Infect Genet Evol. (2014) 21:375–83. 10.1016/j.meegid.2013.12.007 24345519

[7] Dhama K Mahendran M Tiwari R Dayal Singh S Kumar D Singh S. Tuberculosis in birds: insights into the *Mycobacterium avium* infections. Vet Med Int. (2011) 2011:712369. 10.4061/2011/712369 21776352 PMC3135220

[8] Harris NB Barletta RG. *Mycobacterium avium* subsp. paratuberculosis in Veterinary Medicine. Clin Microbiol Rev. (2001) 14:489–512. 10.1128/CMR.14.3.489-512.2001 11432810 PMC88986

[9] Ignatov D Kondratieva E Azhikina T Apt A. *Mycobacterium avium*-triggered diseases: pathogenomics. Cell Microbiol. (2012) 14:808–18. 10.1111/j.1462-5822.2012.01776.x 22348543

[10] Falkinham JO. The changing pattern of nontuberculous mycobacterial disease. Can J Infect Dis. (2003) 14:281–6. 10.1155/2003/323058 18159470 PMC2094944

[11] Haug M Awuh JA Steigedal M Frengen Kojen J Marstad A Nordrum IS. Dynamics of immune effector mechanisms during infection with *Mycobacterium avium* in C57BL/6 mice. Immunology (2013) 140:232–43. 10.1111/imm.12131 23746054 PMC3784169

[12] Saunders BM Dane A Briscoe H Britton WJ. Characterization of immune responses during infection with *Mycobacterium avium* strains 100, 101 and the recently sequenced 104. Immunol Cell Biol. (2002) 80:544–9. 10.1046/j.1440-1711.2002.01121.x 12406388

[13] Florido M Goncalves AS Silva RA Ehlers S Cooper AM Appelberg R. Resistance of virulent *Mycobacterium avium* to gamma interferon-mediated antimicrobial activity suggests additional signals for induction of mycobacteriostasis. Infect Immun. (1999) 67:3610–8. 10377146 10.1128/iai.67.7.3610-3618.1999 PMC116551

[14] Bronte V Brandau S Chen SH Colombo MP Frey AB Greten TF. Recommendations for myeloid-derived suppressor cell nomenclature and characterization standards. Nat Commun. (2016) 7:12150. 10.1038/ncomms12150 27381735 PMC4935811

[15] Damuzzo V Pinton L Desantis G Solito S Marigo I Bronte V. Complexity and challenges in defining myeloid-derived suppressor cells. Cytomet Part B Clin Cytomet. (2015) 88:77–91. 10.1002/cytob.21206 25504825 PMC4405078

[16] Veglia F Perego M Gabrilovich D. Myeloid-derived suppressor cells coming of age. Nat Immunol. (2018) 19:108–19. 10.1038/s41590-017-0022-x 29348500 PMC5854158

[17] Gabrilovich DI Nagaraj S. Myeloid-derived suppressor cells as regulators of the immune system. Nat Rev Immunol. (2009) 9:162–74. 10.1038/nri2506 19197294 PMC2828349

[18] Umansky V Sevko A. Tumor microenvironment and myeloid-derived suppressor cells. Cancer Microenviron. (2013) 6:169–77. 10.1007/s12307-012-0126-7 23242672 PMC3717060

[19] Bogdan C. Regulation of lymphocytes by nitric oxide. Methods Mol Biol. (2011) 677:375–93. 10.1007/978-1-60761-869-0_24 20941622

[20] Bogdan C. Nitric oxide and the immune response. Nat Immunol. (2001) 2:907–16. 10.1038/ni1001-907 11577346

[21] Bogdan C. Nitric oxide synthase in innate and adaptive immunity: an update. Trends Immunol. (2015) 36:161–78. 10.1016/j.it.2015.01.003 25687683

[22] Schleicher U Paduch K Debus A Obermeyer S Konig T Kling JC. TNF-mediated restriction of arginase 1 expression in myeloid cells triggers type 2 NO synthase activity at the site of infection. Cell Rep. (2016) 15:1062–75. 10.1016/j.celrep.2016.04.001 27117406 PMC5065922

[23] Tomioka H Saito H Yamada Y. Characteristics of immunosuppressive macrophages induced in spleen cells by *Mycobacterium avium* complex infections in mice. J Gen Microbiol. (1990) 136:965–73. 10.1099/00221287-136-5-965 2380690

[24] Tomioka H Sato K Maw WW Saito H. The role of tumor necrosis factor, interferon-gamma, transforming growth factor-beta, and nitric oxide in the expression of immunosuppressive functions of splenic macrophages induced by *Mycobacterium avium* complex infection. J Leukoc Biol. (1995) 58:704–12. 10.1002/jlb.58.6.704 7499969

[25] Ellner JJ. Suppressor adherent cells in human tuberculosis. J Immunol. (1978) 121:2573–9. 309907

[26] Edwards CK III Hedegaard HB Zlotnik A Gangadharam PR Johnston RB Jr Pabst MJ Chronic infection due to Mycobacterium intracellulare in mice: association with macrophage release of prostaglandin E2 and reversal by injection of indomethacin, muramyl dipeptide, or interferon-gamma. J Immunol. (1986) 136:1820–7. 3005400

[27] Knaul JK Jorg S Oberbeck-Mueller D Heinemann E Scheuermann L Brinkmann V. Lung-residing myeloid-derived suppressors display dual functionality in murine pulmonary tuberculosis. Am J Respir Crit Care Med. (2014) 190:1053–66. 10.1164/rccm.201405-0828OC 25275852

[28] Tsiganov EN Verbina EM Radaeva TV Sosunov VV Kosmiadi GA Nikitina IY. Gr-1dimCD11b+ immature myeloid-derived suppressor cells but not neutrophils are markers of lethal tuberculosis infection in mice. J Immunol. (2014) 192:4718–27. 10.4049/jimmunol.1301365 24711621 PMC4537794

[29] Obregón-Henao A Henao-Tamayo M Orme IM Ordway DJ. Gr1(int)CD11b(+) Myeloid-derived suppressor cells in *Mycobacterium tuberculosis* Infection. PLoS ONE (2013) 8:e80669. 10.1371/journal.pone.0080669 24224058 PMC3815237

[30] Zietara N Łyszkiewicz M Puchałka J Pei G Gutierrez MG Lienenklaus S. Immunoglobulins drive terminal maturation of splenic dendritic cells. PNAS (2013) 110:2282–7. 10.1073/pnas.1210654110 23345431 PMC3568358

[31] Ruangkiattikul N Nerlich A Abdissa K Lienenklaus S Suwandi A Janze N. cGAS-STING-TBK1-IRF3/7 induced interferon-beta contributes to the clearing of non tuberculous mycobacterial infection in mice. Virulence (2017) 8:1303–15. 10.1080/21505594.2017.1321191 28422568 PMC5711412

[32] Bugl S Wirths S Muller MR Radsak MP Kopp HG. Current insights into neutrophil homeostasis. Ann N Y Acad Sci. (2012) 1266:171–8. 10.1111/j.1749-6632.2012.06607.x 22901268

[33] Wu CF Andzinski L Kasnitz N Kröger A Klawonn F Lienenklaus S. The lack of type I interferon induces neutrophil-mediated pre-metastatic niche formation in the mouse lung. Int J Cancer (2015) 137:837–47. 10.1002/ijc.29444 25604426

[34] Novak R Dabelic S Dumic J. Galectin-1 and galectin-3 expression profiles in classically and alternatively activated human macrophages. Biochim Biophys Acta (2012) 1820:1383–90. 10.1016/j.bbagen.2011.11.014 22155450

[35] Meissner T Eckelt E Basler T Meens J Heinzmann J Suwandi A. The *Mycobacterium avium* ssp. paratuberculosis specific mptD gene is required for maintenance of the metabolic homeostasis necessary for full virulence in mouse infections. Front Cell Infect Microbiol. (2014) 4:110. 10.3389/fcimb.2014.00110 25177550 PMC4132290

[36] Serafini P. Myeloid derived suppressor cells in physiological and pathological conditions: the good, the bad, and the ugly. Immunol Res. (2013) 57:172–84. 10.1007/s12026-013-8455-2 24203443

[37] Thwe PM Amiel E. The role of nitric oxide in metabolic regulation of Dendritic cell immune function. Cancer Lett. (2018) 412:236–42. 10.1016/j.canlet.2017.10.032 29107106 PMC5699934

[38] Fulton SA Reba SM Pai RK Pennini M Torres M Harding CV. Inhibition of major histocompatibility complex II expression and antigen processing in murine alveolar macrophages by *Mycobacterium bovis* BCG and the 19-kilodalton mycobacterial lipoprotein. Infect Immun. (2004) 72:2101–10. 10.1128/IAI.72.4.2101-2110.2004 15039332 PMC375182

[39] Grohmann U Bronte V. Control of immune response by amino acid metabolism. Immunol Rev. (2010) 236:243–64. 10.1111/j.1600-065X.2010.00915.x 20636821

[40] Tian T Woodworth J Skold M Behar SM. *In vivo* depletion of CD11c+ cells delays the CD4+ T cell response to *Mycobacterium tuberculosis* and exacerbates the outcome of infection. J Immunol. (2005) 175:3268–72. 10.4049/jimmunol.175.5.3268 16116218

[41] Srivastava S Ernst JD. Cutting edge: Direct recognition of infected cells by CD4 T cells is required for control of intracellular *Mycobacterium tuberculosis in vivo*. J Immunol. (2013) 191:1016–20. 10.4049/jimmunol.1301236 23817429 PMC3725655

[42] Petrofsky M Bermudez LE. CD4+ T cells but Not CD8+ or gammadelta+ lymphocytes are required for host protection against *Mycobacterium avium* infection and dissemination through the intestinal route. Infect Immun. (2005) 73:2621–7. 10.1128/IAI.73.5.2621-2627.2005 15845464 PMC1087360

[43] Lousada S Florido M Appelberg R. Regulation of granuloma fibrosis by nitric oxide during *Mycobacterium avium* experimental infection. Int J Exp Pathol. (2006) 87:307–15. 10.1111/j.1365-2613.2006.00487.x 16875496 PMC2517369

[44] Ehlers S Reiling N Gangloff S Woltmann A Goyert S *Mycobacterium avium* infection in CD14-deficient mice fails to substantiate a significant role for CD14 in antimycobacterial protection or granulomatous inflammation. Immunology (2001) 103:113–21. 10.1046/j.1365-2567.2001.01214.x 11380699 PMC1783221

[45] Frehel C de Chastellier C Offredo C Berche P. Intramacrophage growth of *Mycobacterium avium* during infection of mice. Infect Immun. (1991) 59:2207–14. 2037382 10.1128/iai.59.6.2207-2214.1991 PMC257991

[46] Agdestein A Johansen TB Kolbjornsen O Jorgensen A Djonne B Olsen I. A comparative study of *Mycobacterium avium* subsp. avium and *Mycobacterium avium* subsp. hominissuis in experimentally infected pigs. BMC Vet Res. (2012) 8:11. 10.1186/1746-6148-8-11 22284630 PMC3296603

[47] Smith D Hansch H Bancroft G Ehlers S. T-cell-independent granuloma formation in response to *Mycobacterium avium*: role of tumour necrosis factor-alpha and interferon-gamma. Immunology (1997) 92:413–21. 10.1046/j.1365-2567.1997.00384.x 9497481 PMC1364145

[48] Hawila E Razon H Wildbaum G Blattner C Sapir Y Shaked Y. CCR5 Directs the mobilization of CD11b(+)Gr1(+)Ly6C(low) polymorphonuclear myeloid cells from the bone marrow to the blood to support tumor development. Cell Rep. (2017) 21:2212–22. 10.1016/j.celrep.2017.10.104 29166611

[49] Xu M Zhao Z Song J Lan X Lu S Chen M. Interactions between interleukin-6 and myeloid-derived suppressor cells drive the chemoresistant phenotype of hepatocellular cancer. Exp Cell Res. (2017) 351:142–9. 10.1016/j.yexcr.2017.01.008 28109867

[50] Yang L Edwards CM Mundy GR. Gr-1+CD11b+ myeloid-derived suppressor cells: formidable partners in tumor metastasis. J Bone Miner Res. (2010) 25:1701–6. 10.1002/jbmr.154 20572008 PMC3153347

[51] Youn JI Nagaraj S Collazo M Gabrilovich DI. Subsets of myeloid-derived suppressor cells in tumor-bearing mice. J Immunol. (2008) 181:5791–802. 10.4049/jimmunol.181.8.5791 18832739 PMC2575748

[52] Raber P Ochoa AC Rodriguez PC. Metabolism of L-arginine by myeloid-derived suppressor cells in cancer: mechanisms of T cell suppression and therapeutic perspectives. Immunol Invest. (2012) 41:614–34. 10.3109/08820139.2012.680634 23017138 PMC3519282

[53] Dreher D Nicod LP. Dendritic cells in the mycobacterial granuloma are involved in acquired immunity. Am J Respir Crit Care Med. (2002) 165:1577–8. 10.1164/rccm.2204010 12070053

[54] Hu CE Gan J Zhang RD Cheng YR Huang GJ. Up-regulated myeloid-derived suppressor cell contributes to hepatocellular carcinoma development by impairing dendritic cell function. Scand J Gastroenterol. (2011) 46:156–64. 10.3109/00365521.2010.516450 20822377

[55] Ostrand-Rosenberg S Sinha P Beury DW Clements VK. Cross-talk between myeloid-derived suppressor cells (MDSC), macrophages, and dendritic cells enhances tumor-induced immune suppression. Semin Cancer Biol. (2012) 22:275–81. 10.1016/j.semcancer.2012.01.011 22313874 PMC3701942

[56] Mondanelli G Bianchi R Pallotta MT Orabona C Albini E Iacono A. A relay pathway between arginine and tryptophan metabolism confers immunosuppressive properties on dendritic cells. Immunity (2017) 46:233–44. 10.1016/j.immuni.2017.01.005 28214225 PMC5337620

[57] Jayaraman P Parikh F Lopez-Rivera E Hailemichael Y Clark A Ma G. Tumor-expressed inducible nitric oxide synthase controls induction of functional myeloid-derived suppressor cells through modulation of vascular endothelial growth factor release. J Immunol. (2012) 188:5365–76. 10.4049/jimmunol.1103553 22529296 PMC3358566

[58] Rath M Muller I Kropf P Closs EI Munder M. Metabolism via arginase or nitric oxide synthase: two competing arginine pathways in macrophages. Front Immunol. (2014) 5:532. 10.3389/fimmu.2014.00532 25386178 PMC4209874

[59] Sharda DR Yu S Ray M Squadrito ML De Palma M Wynn TA. Regulation of macrophage arginase expression and tumor growth by the Ron receptor tyrosine kinase. J Immunol. (2011) 187:2181–92. 10.4049/jimmunol.1003460 21810604 PMC4042865

[60] Gomes MS Florido M Pais TF Appelberg R. Improved clearance of *Mycobacterium avium* upon disruption of the inducible nitric oxide synthase gene. J Immunol. (1999) 162:6734–9. 10352292

[61] Mishra BB Lovewell RR Olive AJ Zhang G Wang W Eugenin E. Nitric oxide prevents a pathogen-permissive granulocytic inflammation during tuberculosis. Nat Microbiol. (2017) 2:17072. 10.1038/nmicrobiol.2017.72 28504669 PMC5461879

[62] Braverman J Stanley SA. Nitric oxide modulates macrophage responses to *Mycobacterium tuberculosis* infection through activation of HIF-1alpha and repression of NF-kappaB. J Immunol. (2017) 199:1805–16. 10.4049/jimmunol.1700515 28754681 PMC5568107

[63] MacMicking JD North RJ LaCourse R Mudgett JS Shah SK Nathan CF. Identification of nitric oxide synthase as a protective locus against tuberculosis. Proc Natl Acad Sci USA. (1997) 94:5243–8. 10.1073/pnas.94.10.5243 9144222 PMC24663

