# Supplementary material for: Presence of Infected Gr-1intCD11bhiCD11cint Monocytic Myeloid Derived Suppressor Cells Subverts T Cell Response and Is Associated With Impaired Dendritic Cell Function in Mycobacterium avium-Infected Mice
Source: Front Immunol. 2018 Oct 16;9:2317. doi: 10.3389/fimmu.2018.02317 (PMC6198055; doi:10.3389/fimmu.2018.02317)
Supplement: Figure S1 — Related to Figures 1, 2: Chronic MAA infection induces accumulation of mycobacteria harboring histiocytic cells in murine spleen. (A) Patterns of serum cytokines and chemokines 5 weeks after infection as determined by multiplex ELISA. Statistics shown represent difference between MAA and MAH infected mice serum cytokines (6 mice were included for the two infected groups and 3 mice in PBS control). (B) Kinetics of MAA proliferation in the liver determined by plating and CFU counting. (C) Numbers of splenic lymphocytes of the indicated cell populations. (D) Flow cytometry of spleen suspensions from MAA or MAH-infected mice showing the percentage of lymphocytes in spleens 5 weeks after infection as determined by flow cytometry. (E) Immuno-histological pictures showing expression of cleaved caspase 3 in spleens of uninfected mice and mice infected with MAA for 7, 21, 28, and 35 days. Caspase 3 positive cells were stained brownish color (Mean ± SEM, *p < 0.05, **p < 0.001, ***p < 0.0001: One way ANOVA). [file Data_Sheet_1.pdf]

Figures S1. Related to Figure 1 and 2.

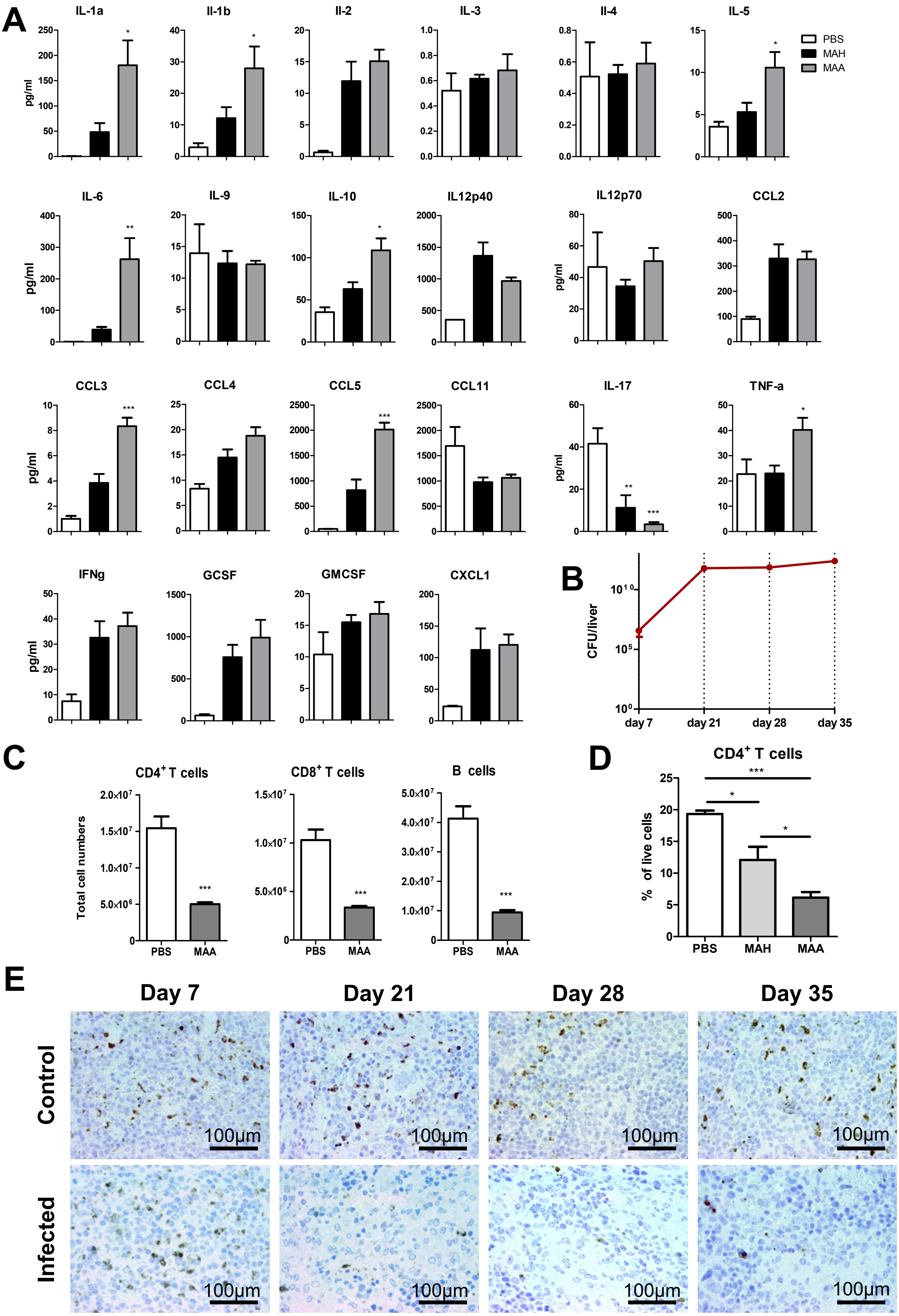

Figure S2 Related to Figure 2

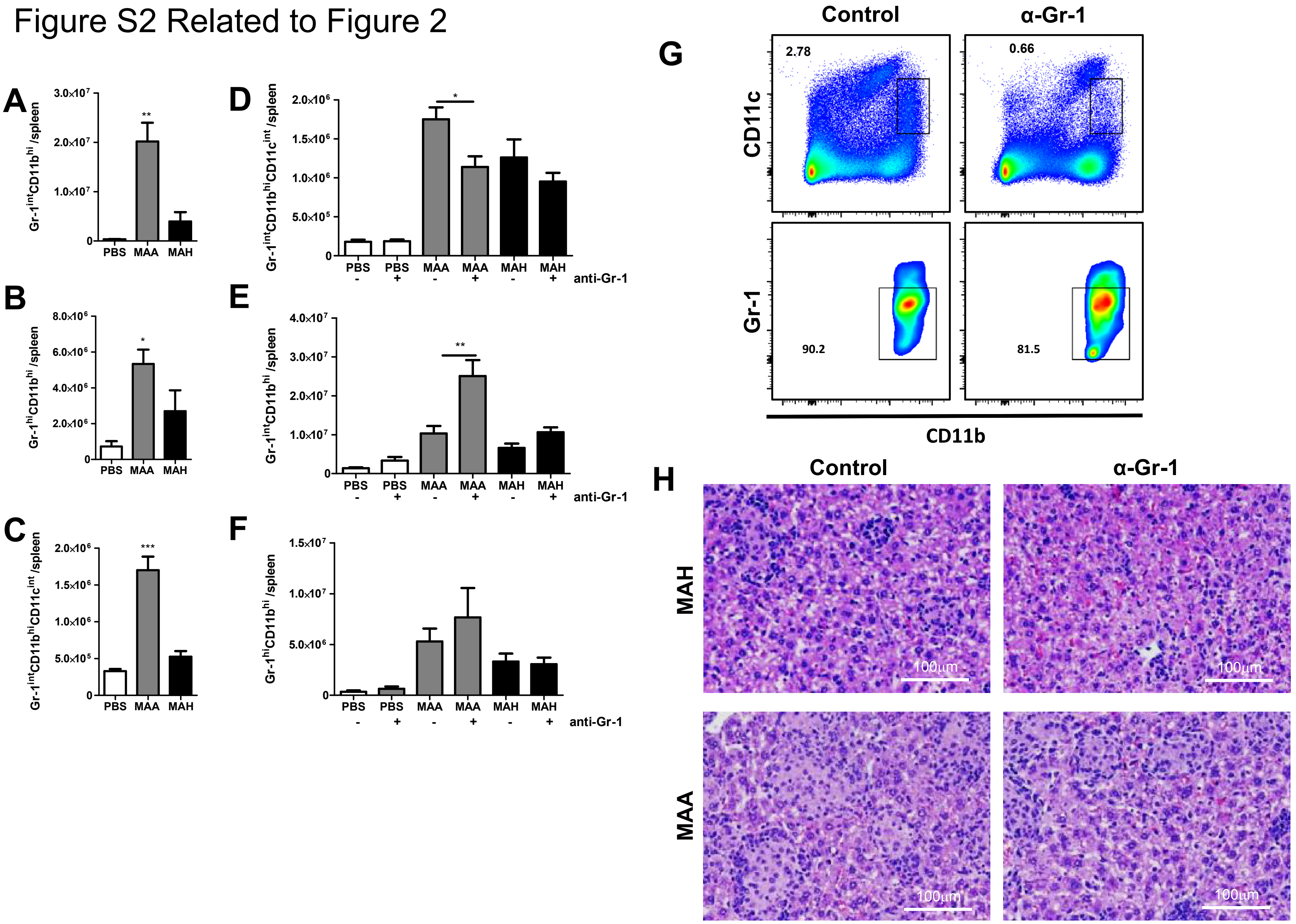

Figures S3. Related to Figure 6.

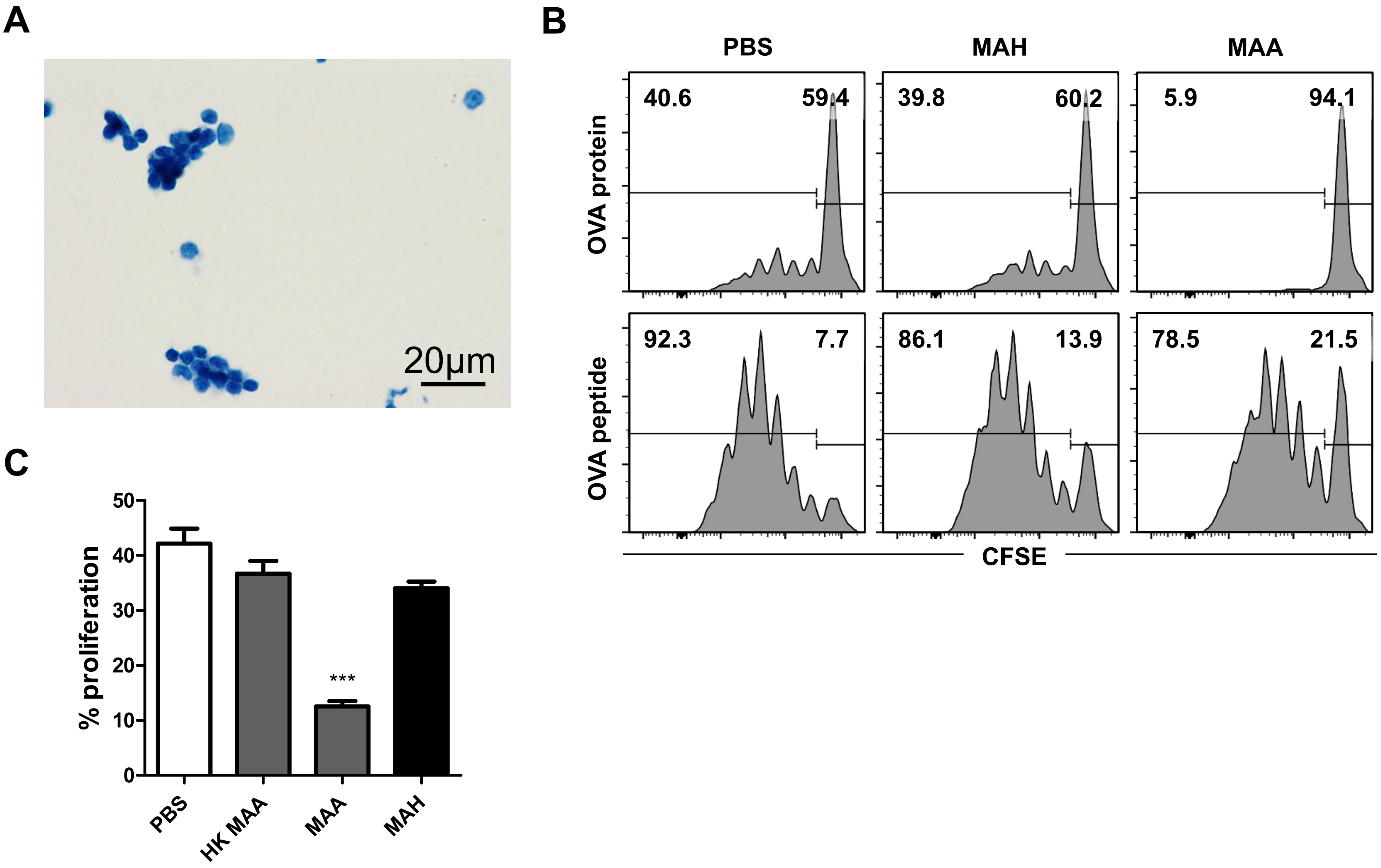

Figure S4. Related to Figure 6.

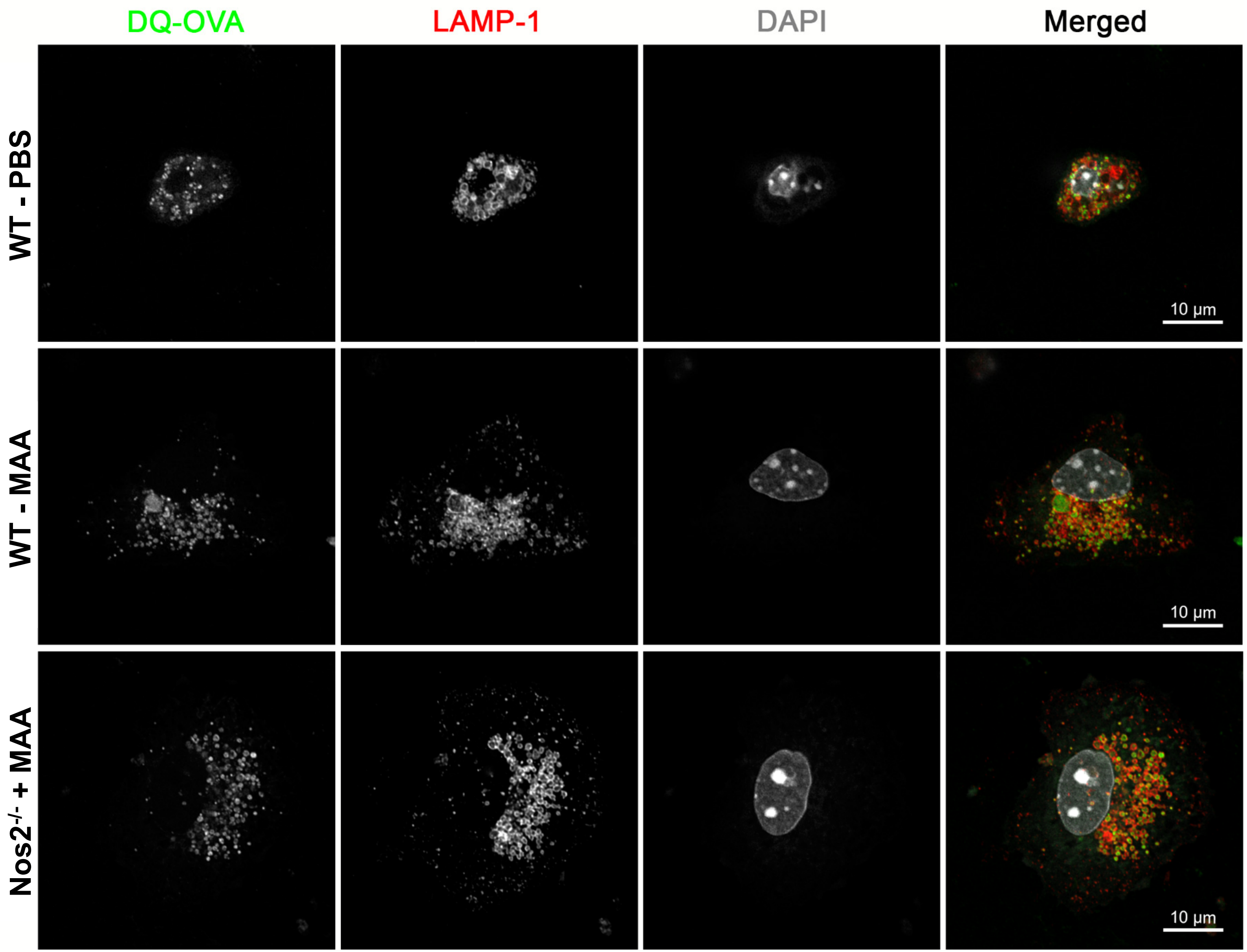

Figure S5

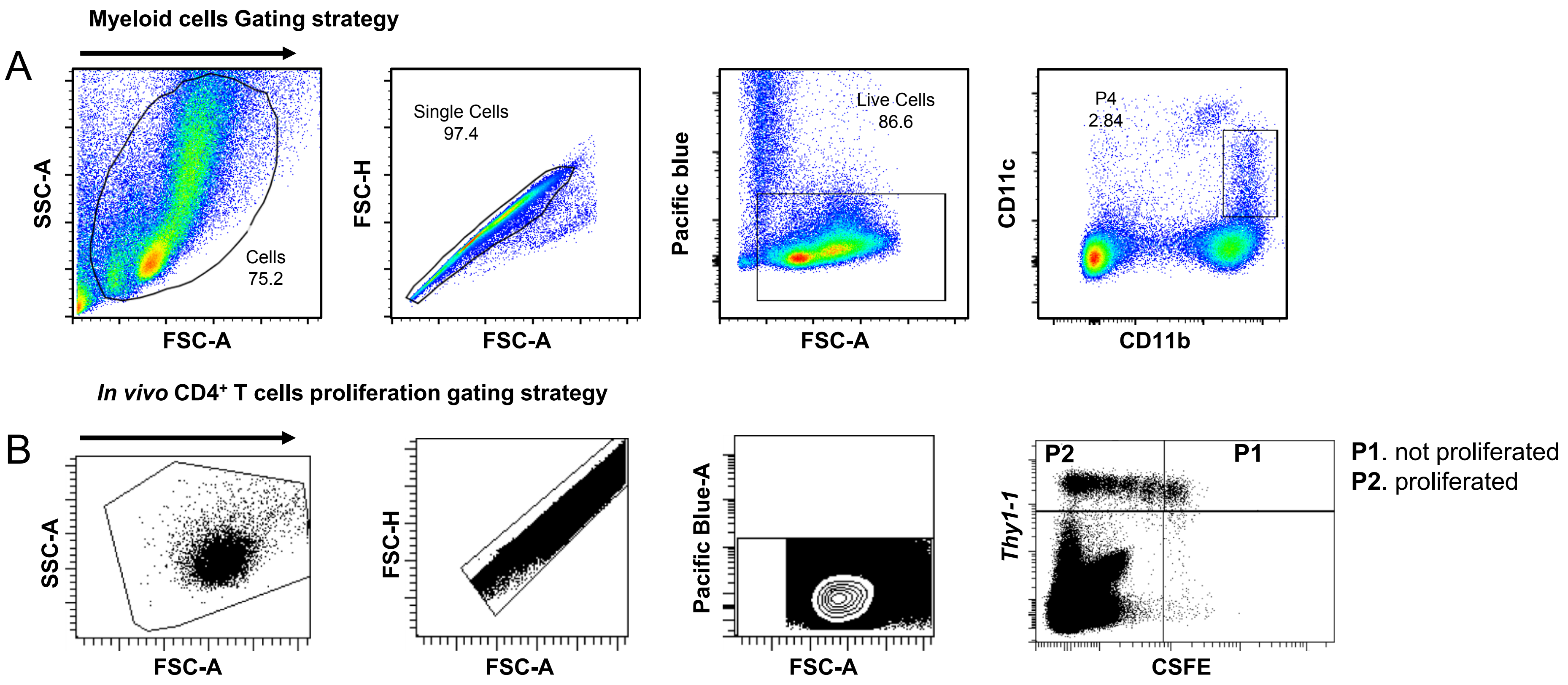
Myeloid cells Gating strategy
A
SSC-A
FSC-A
Cells
75.2
Single Cells
97.4
FSC-H
FSC-A
Live Cells
86.6
Pacific blue
FSC-A
P4
2.84
CD11c
CD11b
In vivo CD4+ T cells proliferation gating strategy
B
SSC-A
FSC-A
FSC-H
FSC-A
Pacific Blue-A
FSC-A
P2
P1
Thy1-1
CSFE
P1. not proliferated
P2. proliferated

Table S1: List of antibodies for flow cytometry used in this study

Antibody	Antigen	Clone	Company
Anti-CD3	CD3 mouse	17A2	eBioscience
Anti-CD4	CD4 mouse	RMA4.5	eBioscience
Anti-CD8	CD8 mouse	53.6.7	eBioscience
Anti-CD11b	CD11b mouse	M1/70	eBioscience
Anti-CD11c	CD11c mouse	N418	eBioscience
Anti-CD25	CD25 mouse	PC61.5	eBioscience
Anti-CD 45RA	CD45RA mouse	3e-F11	BioLegend
Anti-CD 45RB	CD45RB mouse	16A	eBioscience
Anti-CD90.1(Thy1.1)	CD90.1 mouse	HIS51	eBioscience
Anti-Gr-1	Gr-1 mouse	RB6.8C5	BD pharmingen
Anti-Ly6C	Ly6C mouse	AL-21	BD pharmingen
Anti-Ly6G	Ly6G mouse	1A8-Ly6g	eBioscience
Anti-FCR	FCR mouse	2.4G2	BD Pharmingen
Anti-PDL-1	PDL-1 mouse	MIH5	eBioscience
Anti-CD86	CD86 mouse	GL1	eBioscience
Anti-MHC-II	MHC-II mouse	M5/114.15.2	eBiosceince
Anti-CD19	CD19 mouse	1D3	BD pharmingen
Anti-Nos2	Nos2 mouse	CXFNT	eBioscience
Anti-TNF	TNF mouse	MP6-XT22	eBioscience
Anti-LAMP1	Mouse LAMP1	1D4B	Biolegend
